# Ancient marine sediment DNA reveals diatom transition in Antarctica

**DOI:** 10.1038/s41467-022-33494-4

**Published:** 2022-10-02

**Authors:** Linda Armbrecht, Michael E. Weber, Maureen E. Raymo, Victoria L. Peck, Trevor Williams, Jonathan Warnock, Yuji Kato, Iván Hernández-Almeida, Frida Hoem, Brendan Reilly, Sidney Hemming, Ian Bailey, Yasmina M. Martos, Marcus Gutjahr, Vincent Percuoco, Claire Allen, Stefanie Brachfeld, Fabricio G. Cardillo, Zhiheng Du, Gerson Fauth, Chris Fogwill, Marga Garcia, Anna Glüder, Michelle Guitard, Ji-Hwan Hwang, Mutsumi Iizuka, Bridget Kenlee, Suzanne O’Connell, Lara F. Pérez, Thomas A. Ronge, Osamu Seki, Lisa Tauxe, Shubham Tripathi, Xufeng Zheng

**Affiliations:** 1 Institute for Marine and Antarctic Studies (IMAS), Ecology and Biodiversity Centre, University of Tasmania, Hobart, TAS 7004 Australia; 2 Australian Centre for Ancient DNA, Faculty of Sciences, Engineering and Technology, The University of Adelaide, Adelaide, SA 5005 Australia; 3 University of Bonn, Institute for Geosciences, Department of Geochemistry and Petrology, 53115 Bonn, Germany; 4 Lamont-Doherty Earth Observatory, Columbia University, Palisades, NY 10964 USA; 5 British Antarctic Survey, Cambridge, CB3 0ET UK; 6 International Ocean Discovery Program, Texas A&M University, College Station, TX 77845 USA; 7 Dept. of Geography, Geology, Environment Planning, Indiana University of Pennsylvania, Indiana, PA 15705 USA; 8 Faculty of Life and Environmental Sciences, University of Tsukuba, Tsukuba, 305-8572 Japan; 9 ETH Zürich, Geological Institute, Department of Earth Science, 8092 Zürich, Switzerland; 10 Marine Palynology and Paleoceanography, Earth Sciences, Utrecht University, 3584 CB Utrecht, The Netherlands; 11 Scripps Institution of Oceanography, University of California San Diego, La Jolla, CA 92093 USA; 12 Camborne School of Mines, University of Exeter, Cornwall, TR10 9FE UK; 13 Planetary Magnetospheres Laboratory (695), NASA Goddard Space Flight Center, Greenbelt, MD 20771 USA; 14 Department of Astronomy, University of Maryland College Park, College Park, MD 20742 USA; 15 GEOMAR Helmholtz Centre for Ocean Research Kiel, 24148 Kiel, Germany; 16 Earth and Environmental Studies, Montclair State University, Montclair, NJ 07043 USA; 17 Departmento Oceanografia, Servicio de Hidrografia Naval, Buenos Aires, Argentina; 18 State Key Laboratory of Cryospheric Science, Northwest Institute of Eco-Environment and Resources, Chinese Academy of Sciences, Lanzhou, China; 19 Geology Program, University of Vale do Rio dos Sinos, São Leopoldo, RS Brazil; 20 School of Water, Energy and the Environment, Cranfield University, Cranfield, MK43 0AL UK; 21 Cadiz Oceanographic Center (IEO-CSIC), Cadiz, 11006 Spain; 22 College of Earth, Ocean, and Atmospheric Sciences, Oregon State University, Corvallis, OR 97331 USA; 23 College of Marine Science, University of South Florida, St. Petersburg, FL 33701 USA; 24 Earth Environmental Sciences, Korea Basic Science Institute, Cheongju, Republic of Korea; 25 Knowledge Engineering, Tokyo City University, Setagaya-ku, Tokyo, 158-8557 Japan; 26 Department of Earth Sciences, University of California Riverside, Riverside, CA 92521 USA; 27 Department of Earth and Environmental Sciences, Wesleyan University, Middletown, 06459 CT USA; 28 Geological Survey of Denmark and Greenland, Department of Marine Geology, DK-8000 Aarhus C, Denmark; 29 Alfred-Wegener-Institute, Helmholtz Center for Polar and Marine Research, 27570 Bremerhaven, Germany; 30 Institute of Low Temperature Science, Hokkaido University, Sapporo, Hokkaido 060-0819 Japan; 31 Marine Stable Isotope Lab, National Centre for Polar and Ocean Research, Ministry of Earth Sciences, Vasco Da Gama, India; 32 State Key Laboratory of Marine Resource Utilization in South China Sea, Hainan University, 570228 Haikou, Hainan China

**Keywords:** Metagenomics, Palaeoecology, Marine biology, Biodiversity, Palaeoceanography

## Abstract

Antarctica is one of the most vulnerable regions to climate change on Earth and studying the past and present responses of this polar marine ecosystem to environmental change is a matter of urgency. Sedimentary ancient DNA (*sed*aDNA) analysis can provide such insights into past ecosystem-wide changes. Here we present authenticated (through extensive contamination control and *sed*aDNA damage analysis) metagenomic marine eukaryote *sed*aDNA from the Scotia Sea region acquired during IODP Expedition 382. We also provide a marine eukaryote *sed*aDNA record of ~1 Mio. years and diatom and chlorophyte *sed*aDNA dating back to ~540 ka (using taxonomic marker genes SSU, LSU, *psbO*). We find evidence of warm phases being associated with high relative diatom abundance, and a marked transition from diatoms comprising <10% of all eukaryotes prior to ~14.5 ka, to ~50% after this time, i.e., following Meltwater Pulse 1A, alongside a composition change from sea-ice to open-ocean species. Our study demonstrates that *sed*aDNA tools can be expanded to hundreds of thousands of years, opening the pathway to the study of ecosystem-wide marine shifts and paleo-productivity phases throughout multiple glacial-interglacial cycles.

## Abstract (web summary)

Sedimentary ancient DNA can indicate ecosystem-wide changes. Here, the authors show association between warm phases and high diatom abundance in the Antarctic Scotia Sea, in addition to presenting ancient eukaryote sedimentary DNA spanning the last approximately 1 million years.

## Introduction

Polar ecosystems are highly vulnerable to ongoing climate change, and rapidly melting ice-sheets and changes in oceanography and in marine ecosystems are expressed on all levels of the food web^1–3^. Antarctica is arguably the most susceptible polar region to climate, evidenced in the fact that West Antarctica has warmed 2.4 ± 1.2 °C between 1958 and 2010, making it one of the fastest-warming regions globally^4^. Understanding how Southern Ocean organisms respond to climate variability, including throughout past climate shifts, is thus of key importance to predict how the Antarctic marine ecosystem will evolve in the near future.

Sedimentary ancient DNA (*sed*aDNA) analysis studies ancient genetic signals preserved in sediments. Because genetic traces of all organisms, fossilising and soft-bodied, can potentially be preserved in sediment records, the analysis of *sed*aDNA holds enormous potential to go beyond standard environmental proxies and allow reconstruction of entire ecosystems^5,6^. Yet, the recovery of *sed*aDNA is complicated, as only trace-amounts of DNA are preserved and they are fragmented and degraded, which makes *sed*aDNA prone to contamination from modern environmental DNA^5,7^. Recent improvements in *sed*aDNA techniques, including in anti-contamination measures during field work, laboratory work, and the use of bioinformatic DNA damage analysis, now permit authentication of *sed*aDNA detected in sediment samples^6,8–11^.

It is yet to be determined, however, how far back in time marine organisms can be detected using *sed*aDNA tools. So far, the oldest authenticated *sed*aDNA is from ~400,000-year-old terrestrial (cave) sediments^12^, and ~650,000-year-old subarctic permafrost deposits^13^. In polar marine ecosystems, eukaryote *sed*aDNA has been recovered from up to ~140,000-year-old sediments in the Arctic^14–16^ and <25,000-year-old sediments in the Antarctic^7^. Deep polar marine environments are ideal locations for *sed*aDNA research because of favourable DNA preservation^14,15^. They feature constantly low temperatures (~0 °C) and low oxygen (~5 mL L^−1^), and UV radiation is absent^17–19^.

**Fig. 1:**
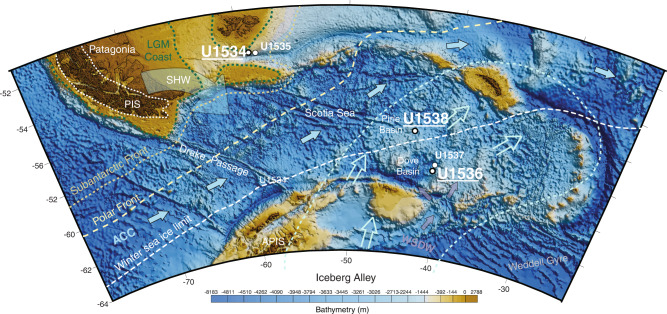
IODP Exp. 382 Site Map. Coring sites at which *sed*aDNA sampling was performed include U1534 (Falkland Plateau), U1536 (Dove Basin) and U1538 (Pirie Basin). Map adapted from IODP^23^ and created using ref. 60.

In 2019, IODP Expedition 382 ‘Iceberg Alley and Sub-Antarctic Ice and Ocean Dynamics’ set out to investigate the long-term climate and oceanographic history of the Antarctic Ice Sheet (AIS). Five sites (U1534–U1538) were drilled east of the Drake Passage, two shelf sites at 53.2°S, the northern edge of the Scotia Sea (U1534, U1535), and three abyssal/deep sites at 57.4°–59.4°S in the Scotia Sea (U1536, U1537, U1538) (Fig. 1). Continuously deposited late Neogene sediments were recovered, which form the basis for ongoing investigations into reconstructing AIS mass loss and associated changes in oceanic and atmospheric circulation^20–23^.

Here, we present a record of metagenomic marine *sed*aDNA from sediments deposited at the northern edge (U1534 - Falkland Plateau) and within the Scotia Sea (U1536 and U1538 – Dove and Pirie Basin, respectively, Fig. 1). Extensive anti-contamination precautions were taken, including clean sampling procedures, the application of chemical tracers to track potential contamination during the coring process^24^, performing laboratory protocols at dedicated ancient DNA facilities, and stringent data filtering combined with *sed*aDNA damage analysis for authenticity assessments of the *sed*aDNA data^25^ (and see Supplementary Information).

## Results

### Core sample contamination assessment via perfluoromethyldecalin (PFMD)

No traces of PFMD were detected at either the periphery (adjacent to core liner) or the centre of the here analysed cores from Sites U1534 and U1536. At Site U1538, PFMD was detected in 21 out of 31 samples from the core periphery (<1.2 ng mL^−1^), and three out of 31 samples from the core centre (<0.64 ng mL^−1^). All PFMD measurements are provided in Supplementary Table 1. Given that PFMD to drill fluid infusion rates were identical at Site U1534 and U1536, and PFMD was detected in samples from these Sites that are not part of this study (Supplementary Data 1), we deem the here analysed samples from U1534 and U1536 free of drill fluid contamination. Likewise, most samples at Site U1538 are considered free of contamination, while the results of three U1538 samples that showed traces of PFMD at the core centre should be interpreted with caution.

### *sed*aDNA read assigned to the three domains using the taxonomic marker genes small (16 S/18S, SSU) and large subunit (23S/28S, LSU) ribosomal RNA SSU and LSU, and both SSU + LSU combined

**Fig. 2:**
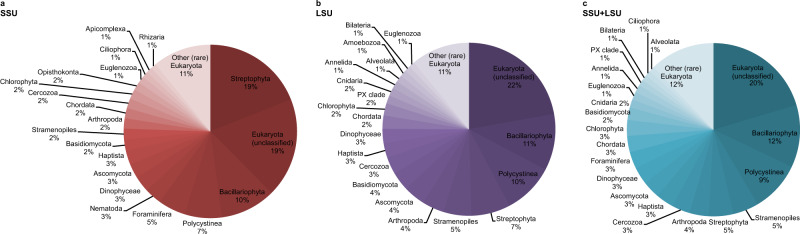
Relative eukaryote abundance across all samples using SSU, LSU and SSU + LSU reference databases. Pie charts were generated based on relative abundances (phylum level) determined after running shotgun data against the SILVA 132 SSU (**a**), LSU (**b**), and combined SILVA SSU + LSU (**c**) databases. Taxa that contributed <1% on average across all samples are summarised as’Other (rare) Eukaryota’. Source data are provided as a Source Data file.

A total of 297,002 reads were assigned to the three domains Bacteria, Archaea, and Eukaryota, using the combined SSU + LSU database (Fig. 2c and see Supplementary Information). When we compared the shotgun data against each of those databases alone, only a total of 142,299 and 189,724 reads were assigned to the three domains using SSU and LSU, respectively, confirming the usefulness of combining the databases prior to alignments (see also Supplementary Data 2). Linear regression analysis between relative abundances per taxon (phylum-level, average across all samples) detected via SSU, LSU and the combined SSU + LSU database showed strong positive relationships between the datasets (*R*^2^_SSU,LSU_ = 0.72, *R*^2^_SSU,SSU+LSU_ = 0.71, *R*^2^_LSU,SSU+LSU_ = 0.90; Supplementary Information Fig. 1), as did Pearson correlation analysis (*p*_SSU,LSU_ = 0.85, *p*_SSU,SSU+LSU_ = 0.84, *p*_LSU,SSU+LSU_ = 0.95; Supplementary Information Table 1), confirming that assignments based on the combined SSU + LSU database were in agreement with assignments based on each database alone. Slightly more taxa were detected using the combined SSU + LSU database (a total of 97 taxa compared to 81 and 84 taxa when using the SSU and LSU reference databases alone, respectively, Fig. 2), thus, we report here on the combined SSU + LSU data.

### *sed*aDNA-derived eukaryote composition at the three sites

At each site, we recovered very few eukaryote reads (0–2) from the mudline samples, maximum number of reads from intermediate depth samples (670 reads at U1534 5.46 mbsf, 7302 reads at U1536 4.46 mbsf, and 22,631 reads at U1538 12.05 mbsf), and few reads in the bottom-most samples (37 reads at U1534, 52 reads in our oldest sample from U1536, and 94 reads from our oldest sample at U1538, dated to ~0.7–2.5 Ma, 1 Ma, and 417 ka, respectively (for details on age determination see Methods section). Across all sites, Eukaryota (category not further classified) were the most abundant (~20% on average across all samples), followed by Bacillariophyta (diatoms, ~11.7%), Polycystinea (which include the Radiolarians, 9.5%), Stramenopiles (not further classified, 4.8%), Streptophyta (4.6%), Arthropoda (3.5%), Cercozoa (3.4%), Haptista (3.4%), Ascomycota (3.25%), Dinophyceae (3.2%), Foraminifera (3.1%), Chordata (3%), Chlorophyta (3%), Basidiomycota (2.3%), Cnidaria (1.5%), Euglenozoa (1.5%), Annelida (1.5%), PX clade (1.1%), Bilateria (1%), Ciliophora (1%) and Alveolata (1%) (Fig. 2). All other phyla contributed <1% on average across all samples to the eukaryote composition (Fig. 2). For details on taxonomy used and assignments see Methods section.

**Fig. 3:**
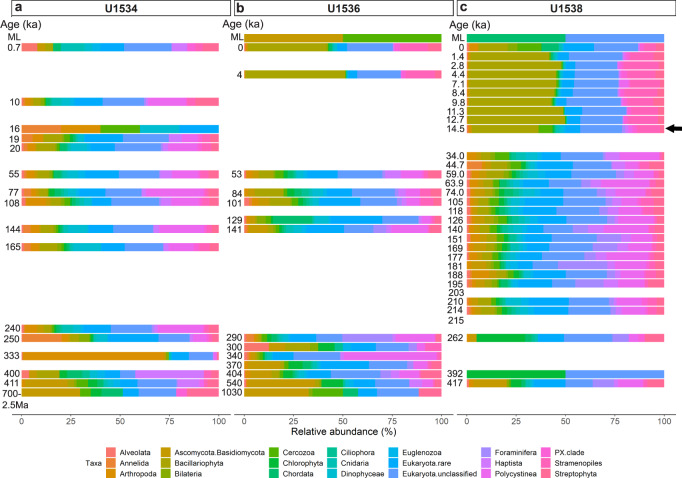
Relative eukaryote abundance at IODP Exp. 382 Sites U1534, U1536, U1538. Relative abundance of eukaryotes is shown at phylum-level as derived from *sed*aDNA at Exp. 382 Sites U1534 (Hole C) (**a**), U1536 (Hole B) (**b**) and U1538 (Holes C and D) (**c**) post combined SSU + LSU alignment. Taxa abundant with at least 1% on average across all samples are shown separately, less abundant taxa are grouped as ‘Eukaryota.rare’. Left axis shows the estimated age (where an age but no bar is shown, no eukaryote reads were identified in that sample). For sample details see Table 1. ML = mudline. The arrow indicates the first sample in which the transition to an increased relative diatom (Bacillariophyta - olive) abundance was detected at Site U1538. Total eukaryote read count: 71,214 reads. Source data are provided as a Source Data file.

Using the SSU + LSU alignments, we identified eukaryotes in all but three samples, and found that eukaryote composition changed with depth at all three sites (Fig. 3). At all sites, Eukaryota (not further classified) contributed most of the assigned reads, followed by diatoms (Bacillariophyta). At Site U1538, the relative abundance of diatoms increased from <10% in sediments as old as 34 ka to ~50% of all eukaryotes in samples younger than 12.7 ka (with ~33% around 14.5 ka; Fig. 3c). The relative abundance of diatoms also increases in younger sediment at Site U1536, however, lower sampling resolution only allows the timing of this shift to be constrained between 53 and 4 ka (Fig. 3b). No obvious increase in the relative abundance of diatoms was observed using *sed*aDNA at Site U1534 (Fig. 3a). While most of our *sed*aDNA samples were younger than 540 ka, we examined the two samples with an estimated age of ~0.7–2.5 Ma (Site U1534) and ~1 Ma (U1536) and acquired few reads (37 and 52, respectively) and few taxa, primarily consisting of Fungi (Asco-, Basidio-, Chytridio- and Mucoromycota), unclassified Eukaryota, and a few rarer groups (e.g., Chordata, Metazoa, Cercozoa, PX clade, Streptophyta and Chlorophyta; Fig. 3a, b and Source Data File).

### *sed*aDNA-derived abundance of photosynthetic organisms

**Fig. 4:**
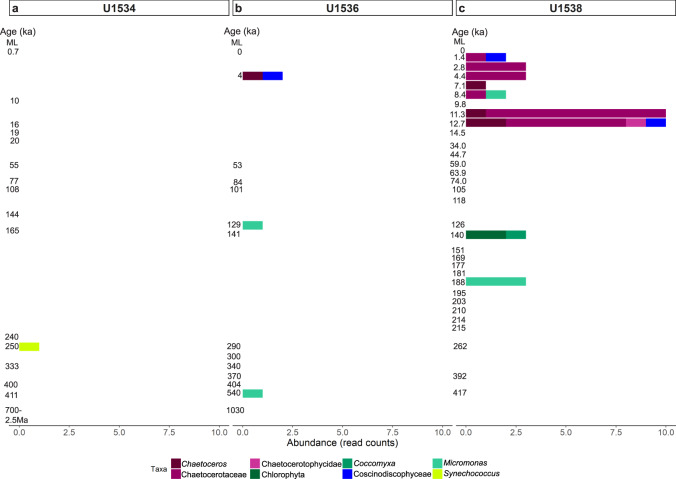
Abundance of photosynthetic organisms at IODP Exp. 382 Sites U1534, U1536, U1538. Abundance of the *psbO* gene (read counts) determined at Exp. 382 Sites U1534 (Hole C) (**a**), U1536 (Hole B) (**b**) and U1538 (Holes C and D) (**c**). Left axis shows the age estimate (where an age but no bar is shown, no *psbO* reads were identified in that sample; for sample details see Table 1). ML = mudline. Figure based on normalised data (subsampled to 1.1Mio reads), total *psbO* read count: 42 reads. Source data are provided as a Source Data file.

We retrieved a total of 42 sequences (131 sequences in the non-subsampled data, see Supplementary Information Fig. 2) of the photosynthesis associated gene *psbO*, which were assigned to *Chaetoceros*, Chatocerotaceae, Chaetocerotophycidae, Coscinodicophyceae (Bacillariophyta); Chlorophyta, *Coccomyxa* and *Micromonas* (Chlorophyta) and *Synechococcus* (Cyanobacteria) (Fig. 4). At Site U1534, one read each was assigned to *Synechococcus*, identified in a sample from intermediate coring depths (~250 ka; Fig. 4a). At Site U1536, one read each was assigned to *Micromonas* (Chlorophyta) in two samples, including the second-oldest sample analysed at this site (~540 ka; Fig. 4b). Chlorophyta (including *Micromonas* and *Coccomyxa*) were detected irregularly throughout the core at Site U1538 (Fig. 4c), including in a ~188 ka sample. Two reads were assigned to diatoms in one of the upper U1536 samples (~4 ka, Fig. 4b), while at U1538 a clear increase in diatom *psbO* reads was detected from 12.7 ka to 11.3 ka (up to 10 reads per sample), after which read numbers decreased again (<3 reads in younger samples; Fig. 4c). Most of these diatom reads were assigned to Chaetocerotaceae (Fig. 4). The sudden increase in the diatom *psbO* gene at Site U1538 around 12.7–11.3 ka indicates increased diatom abundance and possibly intense blooming periods (especially, Chaetocerotaceae), which may have started sometime after 14.5 ka as increased diatom relative abundances determined by SSU + LSU suggests (see previous section).

### Transition to diatom *sed*aDNA at Site U1538 after 14.5 ka

**Fig. 5:**
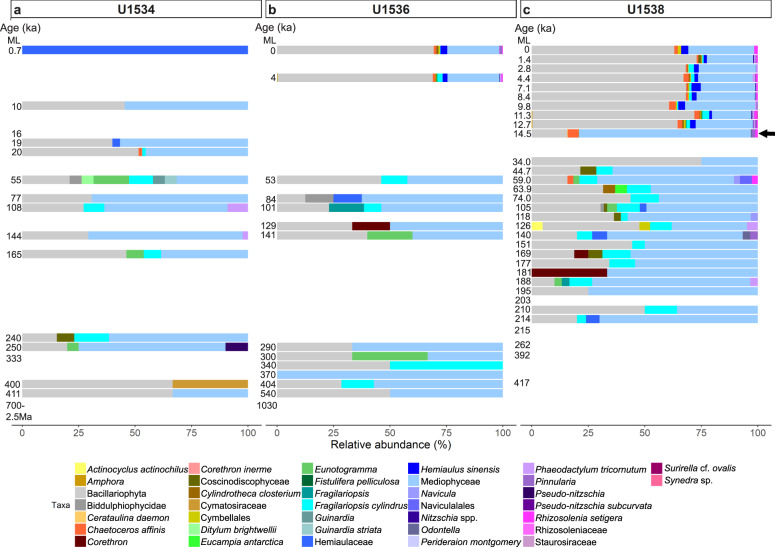
Relative diatom abundance at IODP Exp. 382 Sites U1534, U1536, U1538. Relative abundance of diatoms is shown at lowest taxonomic level resolved from *sed*aDNA at Exp. 382 Sites U1534 (Hole C) (**a**), U1536 (Hole B) (**b**) and U1538 (Holes C and D) (**c**), post combined SSU + LSU alignment. Left axis shows the estimated age (where an age but no bar is shown, no diatom reads were identified in that sample). For sample details, see Table 1. ML = mudline. Arrows indicate the start of increased, consistent presence of *C. affinis* (14.5 ka) and *H. sinensis* (12.7 ka) at Site U1538. Total diatom read count: 27,707 reads. Source data are provided as a Source Data file.

SSU + LSU *sed*aDNA analyses revealed a transition to diatoms dominating the marine eukaryote community at Site U1538 from ~14.5 ka to present, with *psbO* indicating particularly high diatom abundance between 12.7 ka – 11.3 ka (see above, Figs. 3 and 4). To obtain an indication of environmental changes associated with this transition, further investigations were made into the diatom-species detected. Inspection of the diatom *sed*aDNA data derived from the SSU + LSU dataset (total of 27,707 reads, Source Data 4) revealed that, on average across all samples (all sites), most diatom reads were assigned on to the broad group of Bacillariophyceae (not further classified, 47%) and related nodes relatively low taxonomic resolution (Bacillariophyta ~11%, Bacillariophycidae 7%, Bacillariaceae ~2%), Mediophyceae (~23%), *Fragilariopsis cylindrus* (~2.4%), *Chaetoceros affinis* ~1.9% and *Hemiaulus sinensis* (~1.8%; Fig. 5). At Site U1538, Mediophyceae and *F. cylindrus* were at higher relative abundance prior to ~44.7 ka than after, whereas relative abundances of *C. affinis* and *H. sinensis* increased from ~14.5 and ~12.7 ka, respectively, to present. The latter two species were also detected in the two youngest samples at Site U1536 (~4 ka and surface, Fig. 5). Inspection of the reads assigned to these two diatoms in MEGAN CE revealed that while *H. sinensis* reads unambiguously matched modern reference sequences of this diatom and were thus assigned to it based on the LCA algorithm, alternative matches for the same read included *Chaetoceros* spp. Both diatoms (*Hemiaulus, Chaetoceros*) are indicators for neritic, open ocean conditions (see Discussion), with their increased influence evident from ~14.5 ka to present (Fig. 5).

### *sed*aDNA damage analysis

**Fig. 6:**
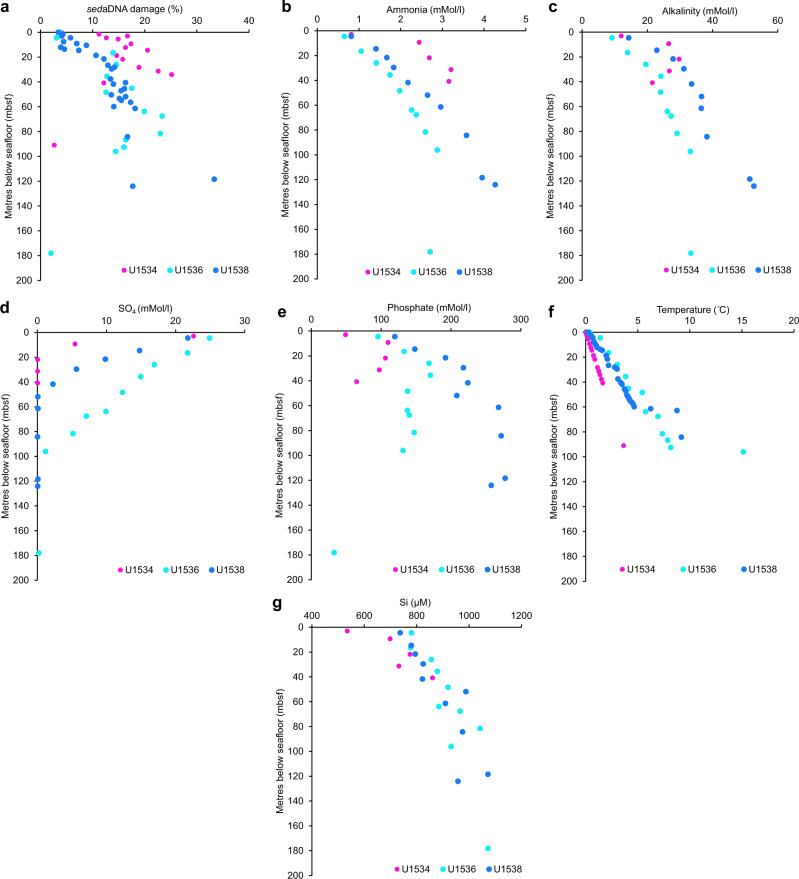
Depth profiles of eukaryote *sed*aDNA damage and geochemical parameters. Plotted against depth (metres below seafloor, mbsf) are **a** the proportion of eukaryote *sed*aDNA damage (based on SSU + LSU alignments), and all geochemical parameters that showed positive correlations with the proportion of eukaryote *sed*aDNA damage, including, **b** ammonia, **c** alkalinity, **d** sulfate, **e** phosphate, **f** temperature and **g** silicon. Source data are provided as a Source Data file.

At Site U1534, 15 ± 6% of the SSU + LSU reads showed *sed*aDNA damage (on average across all samples), while at Site U1536 the damage was 13 ± 7%, and at Site U1538 it was 11 ± 7% (Fig. 6a and Supplementary Data 3). Generally, deeper sediments showed a higher proportion of damage (maximum of 33% at Site U1538 (118.35 mbsf, 392 ka), decreasing towards the surface at all sites (<~3–4% in the shallowest sediments (upper 10 mbsf), Fig. 6a and Supplementary Data 3). In some samples, *sed*aDNA damage deviated from this trend, e.g., 0% *sed*aDNA damage was determined at Site U1538 at 57.55 mbsf (203 ka), although this is based on a single read only (classified as passing our stringent default filtering criteria but not showing damage), and U1534 90.95 mbsf (~0.7–2.5 Ma), where only one ancient read was identified (Supplementary Data 3). The older read identified *Coriolopsis gallica* (Basidiomycetes, Fungi), based on a 56-bp-long sequence assigned to a transcribed RNA sequence (NCBI accession no. GBYM01000074). The remaining reads assigned to eukaryotes in this sample passed our default filtering criteria but did not show damage.

As *psbO* read numbers were very low at all sites, *sed*aDNA damage analysis on the non-subsampled data only achieved results for few taxa and few samples. At Site U1534, U1536 and U1538, 2, 4 and 14 samples showed *sed*aDNA damage, respectively (Supplementary Data 4). The oldest *psbO* genes showing signs of DNA damage were assigned to Haptista (only detected in the non-subsampled data) and Chlorophyta at 240 ka (Site U1534), and to Bacillariophyta at ~4.4 ka (Site U1538, Supplementary Data 4).

### Relationships between *sed*aDNA damage, taxonomic composition and geochemical parameters

Correlation analyses revealed positive relationships between *sed*aDNA damage and ammonia (*r* = 0.56), alkalinity (*r* = 0.52), phosphate (*r* = 0.44), δ^18^O (*r* = 0.32), temperature (*r* = 0.31), silicon (*r* = 0.26), and age of sediments (*r* = 0.21), very weak positive correlations between *sed*aDNA damage with pH (*r* = 0.09), and negative relationships between *sed*aDNA damage with sulfate (*r* = −0.42), and salinity (*r* = −0.15; Supplementary Information Fig. 3 and Supplementary Data 5). This means that *sed*aDNA damage in this study is primarily associated with ammonia, alkalinity and phosphate, i.e., classical indicators for organic matter decomposition (Fig. 6). Correlation analyses between each of the geochemical parameters, as well as benthic δ^18^O (from ref. 26) and the relative abundance of individual eukaryote taxa revealed negative relationships between δ^18^O and diatoms (*r* = −0.41) and positive relationships between δ^18^O and Polycystinea (*r* = 0.42), Dinophycease (*r* = 0.39) Choanoflagellata (*r* = 0.38), Mollusca (*r* = 0.37) and Annelida (*r* = 0.35), meaning that diatoms were associated with warm phases and dinoflagellates, radiolarian, choanoflagellates and Annelida (includes Crustacea) with cold phases (Supplementary Information Fig. 3). Relationships between the remaining geochemical parameters and taxonomic composition was random, and data is provided with Supplementary Information Fig. 3 and Supplementary Data 5.

## Discussion

We investigated marine eukaryote *sed*aDNA acquired during IODP Exp. 382 in the Scotia Sea region. The use of PFMD tracers combined with optimised *sed*aDNA extraction and bioinformatic techniques such as *sed*aDNA damage analyses, allowed us to confirm the presence of at least ~1-milion-year-old marine eukaryote *sed*aDNA generated from metagenomic data. This presents the oldest marine eukaryote *sed*aDNA record to date. Previously, old and authenticated *sed*aDNA records had been from ~400-ka-old cave sediments^12,27^ and ~650-ka-old permafrost deposits^13^. While older claims exist (e.g., chloroplast DNA of 1.4 Ma of age^28^), these have yet to be replicated and authenticated using, for example, metagenomics, or through paired analysis of diagnostic lipid biomarkers as well as geochemical proxies^29,30^. Most of our samples are younger than ~540 ka, and, based on SSU + LSU results, we were able to identify diatom species in samples as old as this (Site U1536), which is, to our knowledge, the oldest diatom record reported from marine *sed*aDNA. Single-copy *psbO* genes of this important phytoplankton group were also identified, confirming peak abundance of diatoms between 12.7 and 11.3 ka at Site U1538, i.e., during the Antarctic Cold Reversal of the last glacial termination. Chlorophyte *psbO* genes were also preserved in samples dating back to ~540 ka (U1536), and, based on SSU + LSU alignments, even in our deepest sample from the early Pleistocene (~0.7–2.5 Ma; U1534).

While the finding of marine eukaryote *sed*aDNA in a sample possibly as old as ~2.5 Ma at Site U1534 is exciting, we consider this result with caution. Firstly, even under ideal conditions (cold, low oxygen, no UV-radiation) ancient DNA, which degrades over time, is not expected to preserve for much longer than ~1 Ma, although reports of non-replicated/authenticated results of ancient bacterial DNA reach back to many millions of years^27^ (and references therein). This could mean that this sample’s true age is rather at the younger end of our age-determination, i.e., ~700 ka, given the stratigraphic uncertainties at Site U1534. Secondly, the taxonomic composition of this sample (and that of the 1 Ma sample at U1536) includes especially fungal groups, suggesting that modern contamination might play a role, as living fungi are a common component of sediment records^31,32^. Possibly, around this age (~1 Ma) the ancient DNA signal fades from the sediment record and DNA from actively living fungi becomes more pronounced. However, the detection of DNA damage in a fungi sequence from the oldest U1534 sample suggests that at least part of the >1 Ma old DNA is of ancient origin. Our finding of marine eukaryote *sed*aDNA in precisely dated samples of Site U1536 are significant for paleoecology, as they expand the temporal window of applying *sed*aDNA analyses as a marine paleo-environmental monitoring tool from ~140 ka to ~1 Ma, i.e., covering multiple glacial-interglacial cycles.

At Site U1538, the *sed*aDNA data reveal a notable transition from a relatively low proportion of diatoms (<10% of eukaryotes) prior to 14.5 ka to a high proportion (~50%) after this date. This timing coincides with well known interhemispheric ice-sheet deglaciation, associated with sea-level rise following Meltwater Pulse 1A at the Boelling/Alleroed transition (~14.5 ka), the spin up of the AMOC and North Atlantic Deep Water (NADW) production, decrease in sea-ice cover and increase in ocean productivity in the Southern Hemisphere^33,34^. Enhanced sea-ice variability and an associated increase in surface marine primary productivity during the Antarctic Cold Reversal (ACR, 14.5–12.7 ka) have also been reported from Patriot Hill Blue Ice records^35^ during this exact same time, and overlap with our finding of high diatom abundance (*psbO*), a highly productive phytoplankton group, between 12.7 and 11.3 ka. After 11.3 ka, relative diatom abundances remain relatively high while total diatom abundance is reduced, which might indicate a re-calibration of the eukaryote marine food web after the peak-productivity phase triggered by the ACR. While community changes were less obvious in samples older than 14.5 ka (possibly a consequence of higher degradation), δ^18^O correlation analyses confirmed an association between increased diatom abundance and past warm phases over the last ~1 Ma.

We showed that, from 14.5 ka to present, the two open ocean diatoms *Chaetoceros affinis* and *Hemiaulus sinensis* increased, at the expense of the sea-ice diatom *Fragilariopsis cylindrus*. *Hemiaulus sinensis* is a relatively rare neritic, warm water/temperate species found along the Argentinian coast^36^, and its detection in sediments as far south as ~59°S in the Scotia Sea is, to our knowledge, a new observation. While it is possible that *H. sinensis* reads were in fact misidentified *Chaetoceros* spp. (Results section), complex northward and southward currents in combination with the Antarctic Circumpolar Current may potentially allow the re-distribution and southward displacement of water-column particles and species^36^, which leaves the possibility of *H. sinensis sed*aDNA indeed being preserved at our study sites. *C. affinis* is commonly considered as an upwelling-indicating, open ocean species^37,38^, while *F. cylindrus* is frequently used as sea-ice proxy^39^. This change in diatom composition is consistent with sea-ice reduction at Site U1538 (Pirie Basin, north of modern winter sea-ice zone) and Site U1536 (Dove Basin, south of modern winter sea-ice zone), starting around 14.5 ka, i.e., following meltwater Pulse 1A^33,34^, and intensifying modern marine conditions ~12.7 ka^35,40^. Neither *C. affinis* nor *H. sinensis* were previously reported as part of the diatom microfossil record at any of the IODP Exp. 382 sites^23^ (noting that detailed microfossil analyses of Exp. 382 sites are still ongoing). It has been shown previously that more fragile, in-situ produced water-column diatoms preserve less well than heavily silicified Antarctic diatoms, thus skewing fossil analyses to the latter species^41^. This underlines the use of *sed*aDNA techniques in complementing microfossil and other paleo-proxy analyses (e.g., biomarkers) for the fine-scale investigation of time periods undergoing environmental change, such as glacial-to-interglacial transitions. Here, the inverse relationship between δ^18^O and relative diatom abundance over the last ~1 Ma indicates that this phytoplankton group played a consistent role throughout major deglaciations in this time frame, with the most obvious change observed at Exp. 382 Scotia Sea sites for the last deglaciation after 14.5 ka.

## Methods

### Sampling location and sediment coring

Samples were collected during IODP Exp. 382 ‘Iceberg Alley and Subantarctic Ice and Ocean Dynamics’ on-board *RV Joides Resolution* between 20 March and 20 May 2019. Specifically, we collected samples at Site U1534 (Falkland Plateau, 606 m water depth), U1536 (Dove Basin, Scotia Sea, 3220 m water depth), and Site U1538 (Pirie Basin, Scotia Sea, 3130 m water depth) (Fig. 1). Site U1534 is located at the Subantarctic Front on a contourite drift at the northern limit of the Scotia Sea. This setting is ideal to study the poorly understood role of Antarctic Intermediate Water (AAIC) and its impact on the Atlantic Meridional Overturning Circulation (AMOC) along the so-called ‘cold water route’ that connects to the Pacific Ocean through the Drake Passage, as opposed to the ‘warm water route’ that connects to the Indian Ocean via the Agulhas Current^42^. Sites U1536 and U1538 are located in the southern and central Scotia Sea, respectively, and were drilled to study the Neogene flux of icebergs through ‘Iceberg Alley’, the main pathway along which icebergs calved from the margin of the AIS travel as they move equatorward into the warmer waters of the Antarctic Circumpolar Current (ACC)^23^. *sed*aDNA samples collected at Site U1534 were from Hole C, at Site U1536 from Hole B, and at Site U1538 from Holes C and D (Table 1), and in the following we refer to site names only. IODP Expedition proposals undergo a rigorous environmental protection and safety review, which is approved by the IODP’s Environmental Protection and Safety Panel (EPSP) and/or the Safety Panel. The same procedure was applied to IODP Exp. 382 and approval was provided by the EPSP. Sediment samples for *sed*aDNA analyses were imported to Australia under Import Permit number 0002658554 provided by the Australian Government Department for Agriculture and Water Resources (date of issue: 19 September 2018), and were stored and extracted at a quarantine approved facility (AA Site No. S1253, Australian Centre for Ancient DNA). No ethical approval was required for this study.

**Table 1:** Sampling location and sample details

U1534C (Falkland Plateau), 53°11.3865′S, 58°45.6296′W, 606.27m	Sample no.	Top depth CSF-A (m)/mbsf	Age (ka)	Glacial (G), interglacial (IG), transition (T)
U1534C-1H-1_MUDLINE	23,006	0	0.7	IG
U1534C-1H-1_0cm	23,005	0	0.7	IG
U1534C-1H-1_145cm	23,008	1.45	10	IG
U1534C-1H-2_145cm	23,011	2.95	16	G
U1534C-1H-3_145cm	23,012	4.45	19	G
U1534C-1H-4_96cm	23,014	5.46	20	G
U1534C-1H_CtrlAir	23,015	–	–	–
U1534C-2H-2_145cm	23,017	9.25	55	T
U1534C-2H-4_145cm	23,018	12.25	77	T
U1534C-2H-6_66cm	23,019	14.46	108	T
U1534C-3H-2_145cm	23,020	18.75	144	G
U1534C-3H-4_145cm	23,021	21.75	165	G
U1534C-4H-2_145cm	23,022	28.25	240	IG
U1534C-4H-4_145cm	23,023	31.25	250	G
U1534C-4H-6_124cm	23,024	34.04	333	IG
U1534C-4H_CtrlAir	23,025	–	–	–
U1534C-5H-2_145cm	23,026	37.75	400	IG
U1534C-5H-4_145cm	23,027	40.75	411	IG
U1534C-10H-6_115cm	23,028	90.95	700ka–2.5 Ma	ND (age uncertainty)
U1534C-10H_CtrlAir	23,029	–	–	–
U1534C_CtrlPFT	23,016	N/A	–	–

U1536B (Dove Basin), 59°26.4608′S, 41°3.6399′W, 3220.06m				
U1536B-1H-1_MUDLINE	23,071	0	0	IG
U1536B-1H-1_0cm	23,072	0	0	IG
U1536B-1H_CtrlAir	23,087	N/A	–	–
U1536B-1H-3_145cm	23,073	4.46	4	IG
U1536B-3H-6_133cm	23,074	16.43	53	G
U1536B-4H-6_136cm	23,075	25.96	84	IG
U1536B-5H-6_145cm	23,076	35.55	101	IG
U1536B-6H-6_145cm	23,077	45.05	129	IG
U1536B-7H-2_145cm	23,078	48.31	141	G
U1536B-8H-6_121cm	23,079	63.81	290	G
U1536B-9H-2_145cm	23,080	67.55	300	IG
U1536B-10H-5_145cm	23,081	81.55	340	G
U1536B-11H-2_145cm	23,082	86.55	370	G
U1536B-11H-6_145cm	23,083	92.55	405	T
U1536B-12H-2_145cm	23,084	96.05	540	G
U1536B-20H-6_141cm	23,085	178.04	1030	IG
U1536B-12H_CtrlAir	23,244	–	–	–
U1536B_CtrlPFT	23,086	–	–	–

U1538 (Pirie Basin), 57°26.5387′S, 43°21.4521′W, 3130.21m				
U1538C-1H-1_MUDLINE	23,136	0	0	IG
U1538C-1H-1_0cm	23,137	0	0	IG
U1538C-1H-1_145cm	23,138	1.45	1.4	IG
U1538C-1H-2_145cm	23,139	2.95	2.8	IG
U1538C-1H-3_145cm	23,140	4.45	4.4	IG
U1538C-2H-1_145cm	23,141	7.55	7.1	IG
U1538C-2H-2_145cm	23,142	9.05	8.4	IG
U1538C-2H-3_145cm	23,143	10.55	9.8	IG
U1538C-2H-4_145cm	23,144	12.05	11.3	IG
U1538C-2H-5_145cm	23,145	13.55	12.7	T
U1538C-2H-6_95cm	23,146	14.55	14.5	T
U1538C-3H-2_145cm	23,147	18.55	34.0	G
U1538C-3H-4_145cm	23,148	21.55	44.7	G
U1538C-4H-1_145cm	23,149	26.55	59.0	G
U1538C-4H-2_145cm	23,150	28.05	63.9	G
U1538C-4H-3_145cm	23,151	29.55	74.0	T
U1538C-5H-2_145cm	23,152	37.55	105	IG
U1538C-5H-4_145cm	23,153	40.55	118	IG
U1538C-5H-5_109cm	23,154	41.69	126	IG
U1538C-6F-1_144cm	23,155	45.54	140	G
U1538C-6F-2_146cm	23,156	47.05	151	G
U1538C-7H-1_145cm	23,157	50.35	169	G
U1538C-7H-2_145cm	23,158	51.85	177	G
U1538C-7H-3_145cm	23,159	53.35	181	G
U1538C-7H-4_145cm	23,160	54.85	188	G
U1538C-7H-5_145cm	23,161	56.35	195	T
U1538C-7H-6_115cm	23,162	57.55	203	IG
U1538C-8H-1_145cm	23,163	59.85	210	IG
U1538C-8H-2_145cm	23,164	61.35	214	IG
U1538C-8H-3_145cm	23,165	62.85	215	IG
U1538C-10H-5_145cm	23,166	84.23	262	G
U1538C-10H_CtrlAir	23,168	–	–	–
U1538C_CtrlPFT	23,167	–	–	–

U1538 (Pirie Basin), 57°26.5335′S, 43°21.4723′W, 3130.4m				
U1538D-14H-1_145cm	23,169	118.35	392	IG
U1538D-14H-5_110cm	23,170	124.04	417	IG

Listed are all *sed*aDNA samples as well as controls collected alongside sediment samples (Air Controls and PFMD (PFT) controls). IODP Expedition 382 Sites (incl. section names), as well as their latitude, longitude and water depth (m) are provided. Top depth CSF-A corresponds to metres below seafloor (mbsf). Ages were assigned based on ref. 45 (Methods section) and glacial/interglacial assignments were based on refs. 26, 45.

### Sample age determination

Age control for Site U1534 is based on tuning of benthic foraminifera δ^18^O to the LR04 stack^43^. Wherever present specimens of *Uvigerina bifurcata* were picked from samples at 10 cm intervals. During warmer periods when *U. bifurcata* was not present, *Melonis affinis* and/or *Hoeglundina elegans* were analysed. Sedimentation rates over the intervals sampled for *sed*aDNA typically range between 6 and 30 cm/kyr, with rates exceeding 100 cm/kyr during the Last Glacial Maximum ~20,000 years ago (20 ka). For our deepest sample, U1534C-10H-6_115cm (90.95 mbsf), we only have biostratigraphically assigned ages available (shipboard data), which date this sample as early Pleistocene (~2.5–0.7 million years ago, Ma^44^).

Low-resolution age control for both Sites U1536 and U1538 was established using shipboard magneto- and biostratigraphy^21,23^. Average sedimentation rates are ~10 cm/kyr for Site U1536, with elevated values (up to 20 cm/kyr) in the upper ~80 mbsf (the last ~400 ka). Site U1538 average sedimentation rates are twice as high, averaging ~20 cm/kyr. Especially in the upper ~430 mbsf (the last 1.8 Ma), rates are up to 40 cm/kyr. Higher resolution age models are based on dust climate couplings, correlating sedimentary dust proxy records such as magnetic susceptibility and sedimentary Ca and Fe records to ice-core dust proxy records over the last 800 ka^45^ and to a benthic isotopic stack^26^ before that. These age models were established for Site U1537 (adjacent to Site U1536) and provide orbital to millennial scale resolution. For this study we correlated sedimentary cycles of Sites U1536 and U1538 to U1537 to achieve similar resolution and to be able to determine if a sample originates from a glacial or interglacial period (Table 1).

### Sampling of *sed*aDNA

A detailed description of *sed*aDNA sampling methods can be found in ref. 24. In brief, we used advanced piston coring (APC) to acquire sediment cores, which recovers the least disturbed sediments^46–48^ and is thus the preferred technique for *sed*aDNA sampling. All samples were taken on the ship’s ‘catwalk’, where, once the core was on deck, the core liners were wiped clean twice (3% sodium hypochlorite, ‘bleach’) at each cutting point. Core cutting tools were sterilised before each cut (3% bleach and 80% ethanol) of the core in 1 m sections. The outer ~3 mm of surface material were removed from the bottom of each core section to be sampled, using sterilised scrapers (~4 cm wide; bleach and ethanol treated). A cylindrical sample was taken from the core centre using a sterile (autoclaved) 10 mL cut-tip syringe, providing ~5 cm^3^ of sediment material. The syringe was placed in a sterile plastic bag (Whirl-Pak) and immediately frozen at −80 °C. The mudline (sediment/seawater interface) was transferred from the core liner into a sterile bucket (3% bleach treated), and 10 mL sample was retained in a sterile 15 mL centrifuge tube (Falcon) and frozen at −80 °C. Samples were collected at various depth intervals depending on the site to span the Holocene up to ~1 million years (Table 1). This lower depth/age limit was determined by switching coring system from APC to the extended core barrel (XCB) system.

To test for potential airborne contamination, at least one air control was taken during the *sed*aDNA sampling process per site. For this, an empty syringe was held for a few seconds in the sampling area and then transferred into a sterile plastic bag and frozen at −80 °C. The air controls were processed, sequenced and analysed alongside the sediment samples.

### Contamination control using perfluoromethyldecalin tracers

As part of the APC process, drill fluid (basically, seawater) is pumped into the borehole to trigger the hydraulic coring system, therefore, the potential for contamination exists due to drill fluid making contact with the core liner. To assess the latter, we added the non-toxic chemical tracer perfluoromethyldecalin (PFMD) to the drill fluid at a rate of ~0.55 mL min^−1^ for cores collected at Sites U1534 and U1536^49^. As we found that PFMD concentrations were very low at these sites (Results section), the infusion rate was doubled prior to *sed*aDNA sampling at Site U1538 to ensure low PFMD concentrations represent low contamination and not delivery failure of PFMD to the core. At each *sed*aDNA sampling depth, one PFMD sample was taken from the periphery of the core (prior to scraping, to test whether drill fluid reached the core pipe), and one next to the *sed*aDNA sample in the centre of the core (after scraping, to minimise differences to the *sed*aDNA sample, and testing if drill fluid had reached the core centre). We transferred ~3 cm^3^ of sediment using a disposable, autoclaved 5 mL cut-tip syringe into a 20 mL headspace vial with metal caps and Teflon seals. We also collected a sample of the tracer-infused drill fluid at each site, by transferring ~10 mL of the fluid collected at the injection pipe on the rig floor via a sterile plastic bottle into a 15 mL centrifuge tube (inside a sterile plastic bag) and freezing it at −80 °C. These drill fluid controls were processed and analysed in the same way as the *sed*aDNA samples including sequencing. Samples were analysed using gas chromatography (GC-µECD; Hewlett-Packard 6890).

A detailed description of the PFMD GC measurements is provided in ref. 24. Briefly, PFMD measurements were undertaken in batches per site for U1534, U1536 and U1538. This included the analyses of PFMD samples collected at two additional holes at these sites, U1534D and U1536C, from which we also collected *sed*aDNA samples but that are not part of this study. PFMD is categorised as the stereoisomers of PFMD (C_11_F_20_), which add up to 87-88% (and with the remaining 12% being additional perfluoro compounds unable to be separated by the manufacturer). We exclusively refer to the first and measurable PFMD category, calibrating for the 88% in bottle concentrations during concentration calculations. Each GC analysis run included the measurement of duplicate blanks and duplicate PFMD standards. Due to a large sample number, PFMD at Site U1538 was measured in three separate runs, with the first and last run including triplicate blank and triplicate PFMD standards (duplicates in the second run), and the last run also containing a drill-fluid sample. To blank-correct PFMD concentrations, we subtracted the average PFMD concentration of all blanks per run from PFMD measurements in that run. To determine the detection limit of PFMD, we used three times the standard deviation of the average blank PFMD values per run; due to all blank values for the U1538 runs being 0, we used three times the standard deviation of the lowest PFMD standard for this site in this calculation. This provided us with a PFMD detection limit of 0.2338 ng mL^−1^. Any PFMD measurements of samples below this limit were rejected.

### *sed*aDNA extractions and metagenomic library preparations

A total of 80 *sed*aDNA extracts and metagenomic shotgun libraries (Table 1) were prepared following^8,10^. For the *sed*aDNA extractions, we randomised our samples and controls and extracted *sed*aDNA in batches of 16 extracts/libraries at a time, with each batch including at least one air control and one extraction blank control (EBC), and the last batch including mudline and PFMD samples to avoid contamination of the *sed*aDNA samples. In brief, we used 20 µL *sed*aDNA extracts in a repair reaction (using T4 DNA polymerase, New England Biolabs, USA; 15 min, 25 °C), then purified the *sed*aDNA (MinElute Reaction Cleanup Kit, Qiagen, Germany), ligated adaptors (T4 DNA ligase, Fermentas, USA, where truncated Illumina-adaptor sequences containing two unique 7 base-pair (bp) barcodes were attached to the double-stranded DNA; 60 min, 22 °C), purified the *sed*aDNA again (MinElute Reaction Cleanup Kit, Qiagen), and then added a fill-in reaction with adaptor sequences (Bst DNA polymerase, New England Biolabs, USA; 30 min, 37 °C, with polymerase deactivation for 10 min, 80 °C). We amplified the barcoded libraries using IS7/IS8 primers^50^ (8 replicates per sample, where each replicate was a 25 µL reaction containing 3 µL DNA template; using 22 cycles), purified (AxyPrep magnetic beads, Axygen Biosciences, USA; 1:1.8 library:beads) and quantified them (Qubit dsDNA HS Assay, Invitrogen, Molecular Probes, USA). We amplified the libraries (8 replicates per sample, 13 amplification cycles) using IS4 and GAII Indexing Primers^50^, purified (AxyPrep magnetic beads, at a ratio of 1:1.1 library:beads), quantified and quality-checked using Qubit (dsDNA HS Assay, Invitrogen, USA) and TapeStation (Agilent Technologies, USA). We combined the libraries into an equimolar pool (volume of 68 µL in total), diluted this pool with nuclease-free H_2_O to 100 µL, and performed a ‘reverse’ AxyPrep clean-up to retain only the small DNA fragments typical for ancient DNA (≤ 500 bp; initial library:beads ratio of 1:0.6, followed by 1:1.1, and double-eluted in 30 µL nuclease-free H_2_O^8,51^). We added one more AxyPrep clean-up to remove primer-dimer (library:beads ratio of 1:1.05) and checked *sed*aDNA quantity and quality via TapeStation and qPCR (QuantStudio, Applied Biosystems, USA). The libraries sequenced at the Garvan Institute for Medical Research, Sydney, Australia (Illumina NovaSeq 2 × 100 bp).

### *sed*aDNA data processing

The sequencing data was processed and filtered as described in detail in refs. 8, 10. Briefly, data filtering involved the removal of sequences <25 bp (AdapterRemoval v. 2.1.7-foss-2016a, Schubert et al., 2016), removal of low-complexity (Komplexity software^52^,–threshold 0.55) and duplicate reads (‘dedupe’ tool in BBMap v.37.36.), and quality control after each step (FastQC v.0.11.4, Babraham Bioinformatics; MultiQC v.1.0.dev0^53^).

Using MALT (version 0.4.0; semiglobal alignment^54^), we ran our data against different curated reference databases that are commonly used to identify marine eukaryotes, including the SILVA small (16S/18S, *SSU*) and large subunit (23S/28S, *LSU*) ribosomal RNA database (version NR 132, https://www.arb-silva.de/), as well as a combined SSU + LSU database. For the latter, we merged the initial SSU and LSU databases (using the ‘cat’ command) into one database prior to the alignments, with the intention to maximise taxonomic resolution and number of reads (as has been shown before by using both SILVA SSU and LSU^7^, see Supplementary Information and Supplementary Data 2). To further maximise the number of reads (to enable robust downstream and *sed*aDNA damage analysis, see below), we avoided normalising (rarefying) our SSU/LSU data and worked with relative abundances. For an estimate of total abundance of phytoplankton, we also ran our data against a recently developed database for the single-copy photosynthetic gene *psbO*, which is present in both prokaryotes and eukaryotes, mainly in one copy per genome^55^. The latter was initially performed with non-subsampled (non-rarefied) data to be able to determine an adequate subsampling depth^56^ (representative of the diversity in the data) for subsequent quantitative analyses (excluding potential artifacts due to differences in library sizes). We determined 1.1 Mio reads as an adequate subsampling depth (lowest number of reads in libraries that contained *psbO*, see Supplementary Information), and subsampled all samples to this depth for quantitative *psbO* analyses. The resulting.blastn files (SSU + LSU, *psbO*) were converted to .rma6 files using the Blast2RMA tool in MEGAN (version 6_18_9), with the default settings except for a minimum support percent of zero (‘off’) and a minimum percent identity of 95%.

Subtractive filtering (i.e., subtracting reads for species identified in EBCs, air, and PFMD controls from samples) was conducted with MEGAN CE^57^ (version 6.21.12). The filtered read counts per taxon (eukaryotes only and phylum-level for SSU and LSU datasets, as well as species level for diatoms to investigate this class in more detail, see Results; and species level (all species) for the *psbO* dataset) and site were exported for downstream analysis. All taxa determined in the controls post-SSU + LSU alignments are listed in Supplementary Data 6; no contaminants were detected in the *psbO* dataset. We used the default settings for analysing the taxonomic data in MEGAN CE, i.e., following the NCBI taxonomy and using a LCA (lowest common ancestor) approach, where every read is assigned to a taxon and if a read has significant matches to two different taxa a and b, where a is an ancestor of b, then only the more specific match to b will be used^57^. Where a taxon was determined multiple times with different reference-groups (e.g., ‘Stramenopiles’, ‘Stramenopiles incertae sedis’ ‘Stramenopiles environmental sample’, Stramenopiles unclassified’) then they were grouped (e.g., ‘Stramenopiles’). For visualisation purposes of the large SSU-LSU phylum-level dataset, we kept all taxa that were abundant with at least 1% on average across all samples as separate taxa (combining the two fungal groups Ascomycota and Basidiomycota), while taxa less abundant than that were grouped (‘Eukaryota.rare’; Results section, Fig. 3). To facilitate visualisation of the SSU-LSU derived diatom dataset, we grouped broader taxa (i.e., ‘Bacillariophyta’, ‘Bacillariophyceae’, ‘Bacillariophycidae’, ‘Bacillariaceae’ as ‘Bacillariophyta’, and ‘Coscinodiscophyceae”, Coscinodiscophycidae’, ‘Coscinodiscales’, ‘Coscinodiscaceae’ as ‘Coscinodiscophyceae’ (Results section, Fig. 5).

### *sed*aDNA damage analysis

As a means of authenticating our *sed*aDNA, we tested *sed*aDNA damage using the ‘MALTExtract’ and ‘Postprocessing’ tools of the HOPS v0.33-2 pipeline^25^. We used the same configurations as in Armbrecht et al. (2021), using the taxalist ‘Eukaryota’ (i.e., only specifying the term ‘Eukaryota’, which captures all eukaryotes) for the SSU + LSU dataset, and a taxalist containing all taxa identified via the non-subsampled *psbO* data, see Supplementary Information) for the *psbO* dataset. We used the non-subsampled *psbO* data because the subsampled data provided too few reads (<50^25^, Results section) for damage analysis. MaltExtract outputs a read summary, with reads are categorised as ancient (showing damage) or default (passing stringent filtering criteria but not showing damage) for the taxalist-specified taxa^25^. Based on the latter (ancient vs. default reads), the proportion of *sed*aDNA damage per taxon was determined for each dataset (SSU + LSU and non-subsampled *psbO*). A summary of reads classified as ancient and default are provided with the Supplementary Data 3 and 4.

### Statistics and correlations

To ensure that our new combined SSU + LSU database generates comparable taxonomic composition to SSU and LSU alone, we performed regression and correlation analyses on the average relative abundance per taxon (phylum-level) acquired when using the SSU, the LSU and the combined SSU + LSU database (using the software PAST Software v.4.03^58^; and with results provided in Supplementary Information Fig. 1, Supplementary Information Table 1).

Lastly, we investigated potential relationships between *sed*aDNA damage (determined by HOPS post SSU-LSU alignment), relative abundances of eukaryotes post SSU + LSU alignment (phylum-level) and downcore temperature and porewater geochemical parameters, which were measured as part of the shipboard sampling during IODP Exp. 382. Downhole formation temperatures were calculated based on the temperature gradient obtained at each Site with the Advanced Piston Corer Temperature Tool (APCT)^24^. Key geochemical parameters for the assessment of organic matter degradation rates (ammonium, alkalinity, phosphate, sulfate, mMol L^−1^), and total silicon (µM), were routinely determined for each site for the characterisation regarding the intensity of redox zonation in each core^24^. To investigate relationships between taxonomic composition and cold and warm climate phases, we added benthic δ^18^O data from^26^ corresponding to the ages assigned to our samples to this correlation analysis. Mudline samples, as well as the two samples 23162 (U1438 57.55 mbsf) and 23165 (U1538 62.85 mbsf), were removed from the following analyses as no eukaryotes were determined in these samples. Pearson correlation analysis was performed in PAST v.4.03^58^.

### Reporting summary

Further information on research design is available in the Nature Research Reporting Summary linked to this article.

## Supplementary information

Supplementary Information

Description of Additional Supplementary Files

Supplementary Data 1 -6

Reporting Summary

## Data Availability

The following databases were used in this study: SILVA small (version 132Ref-nr) and large (version 132Ref) subunit ribosomal RNA database (https://www.arb-silva.de/), and *psbO*^55^ (https://www.ebi.ac.uk/biostudies/studies/S-BSST659?query=S-BSST659). Detailed Supplementary Information on methods and analysis is provided with this submission. The demultiplexed raw sequencing data generated and analysed during this study have been deposited in the NCBI Sequence Read Archive database (https://www.ncbi.nlm.nih.gov/sra) under Accession code/BioProject PRJNA861836 (BioSamples SAMN29928044 - SAMN29928123)^59^, and includes metadata for each sediment and control sample. For further requests please contact the corresponding author. Source data are provided with this paper.

## Source data

Source Data

## Code availability

The bioinformatic pipeline used in this study was previously published in ref. 8 and additional information is provided with the Supplementary Information Notes 1–3.
