# Supplementary material for: Ancient marine sediment DNA reveals diatom transition in Antarctica
Source: Nat Commun. 2022 Oct 2;13:5787. doi: 10.1038/s41467-022-33494-4 (PMC9527250; doi:10.1038/s41467-022-33494-4)
Supplement: Supplementary file 1 — Supplementary Information [file 41467_2022_33494_MOESM1_ESM.pdf]

Supplementary Information

Ancient marine sediment DNA reveals diatom transition in Antarctica

Authors: Linda Armbrecht*, Michael E. Weber, Maureen E. Raymo, Victoria L. Peck, Trevor Williams, Jonathan Warnock, Yuji Kato, Iván Hernández-Almeida, Frida Hoem, Brendan Reilly, Sidney Hemming, Ian Bailey, Yasmina M. Martos, Marcus Gutjahr, Vincent Percuoco, Claire Allen, Stefanie Brachfeld, Fabricio G. Cardillo, Zhiheng Du, Gerson Fauth, Chris Fogwill, Marga Garcia, Anna Glüder, Michelle Guitard, Ji-Hwan Hwang, Mutsumi Iizuka, Bridget Kenlee, Suzanne O'Connell, Lara F. Pérez, Thomas A. Ronge, Osamu Seki, Lisa Tauxe, Shubham Tripathi, Xufeng Zheng; *Corresponding author email: linda.armbrecht@utas.edu.au

Table of contents

**Content**	**Page**
Supplementary Notes 1: Comparison of alignments with SILVA SSU, LSU and combined SSU+LSU reference databases	1
Supplementary Fig. 1: Linear regression (SSU, LSU, SSU+LSU)	2
Supplementary Table 1: Correlation analysis statistics (SSU, LSU, SSU+LSU)	2
Supplementary Notes 2: *psbO* analysis	3
Supplementary Fig. 2: Abundance of photosynthetic organisms at IODP Exp. 382 Sites U1534, U1536, U1538 (non-standardised).	3
Supplementary Notes 3: Correlation analyses of *seda*DNA damage, taxonomic composition, and geochemical parameters	4
Supplementary Fig. 3: Correlation analysis plot	5
Supplementary References	6

Supplementary Notes 1: Comparison of alignments with SILVA SSU, LSU and combined SSU+LSU reference databases

It has been shown previously that using a combination of both the small (16S/18S, *SSU*) and large subunit (23S/28S, *LSU*) ribosomal RNA taxonomic marker genes to identify marine eukaryotes from metagenomic *seda*DNA provides a better taxonomic resolution relative to when only one database is used. Specifically, the SSU is better suited for detecting major marine groups such as tintinnids (a group of ciliates), cnidarians, molluscs, and fish, the LSU provided better resolution for crustaceans (e.g., copepods) and haptophytes (e.g., Phaeophyceae), and using a combination of SSU and LSU databases provides a better species resolution for eukaryotes (i.e., an increase in the number of eukaryotic taxa detected) relative to single markers (nearly double the number of taxa compared to using SSU and LSU alone[1]).

Post filtering (removal of short (<25bp), complexity-filtering and deduplication), we retrieved a total of 167.8 Mio reads with an average length of 64 bp for samples and 38 bp for controls (MultiQC[2]). These were aligned with each database (SILVA SSU, LSU and combined SSU+LSU, https://www.arb-silva.de/), which provided a total of 142,299, 189,724, and 297,002 reads assigned to the three domains Bacteria, Archaea, and Eukaryota (for SSU, LSU and SSU+LSU respectively, see Main Text). Next, we exported the read counts data from MEGAN CE[3] (v.6.21.12) for each dataset (SSU, LSU, SSU+LSU; eukaryotes on phylum level), converted to relative abundances and determined the average across all samples (Main Text Fig. 2). We worked with relative abundances in order to retain the maximum number of reads for downstream analyses, providing the total number of reads for completeness (see Main Text Figs. 3 – 5 and associated Source Data). While relative abundances of taxa were very similar between the datasets, slightly more taxa were detected using the combined SSU+LSU database (a

total of 97 taxa compared to 81 and 84 when using the SSU and LSU reference databases alone, respectively (phylum level). In cases where a taxon was detected by either the SSU or the LSU database when using these databases separately, it was always detected via the combined database (Supplementary Data 2). Five taxa were missing in the LSU database and thus could only be detected via the SSU or SSU+LSU database (Supplementary Data 2). Performing a linear Regression Analysis (Excel), and Pearson Correlation Analysis (PAST software v.4.03[4]) on the relative abundances per taxon determined by SSU, LSU and combined SSU+LSU showed strong positive relationships between the datasets (Supplementary Fig. 1, Supplementary Data 2). As such and due to the slightly better taxonomic resolution, we report the taxonomic profiles generated by comparing our shotgun data to the combined SSU+LSU database in the Main Text.

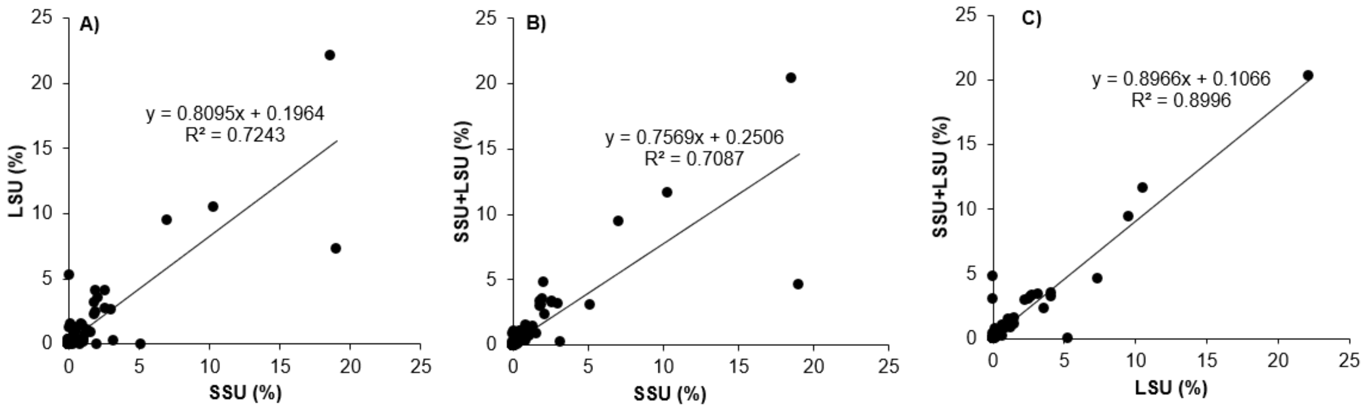

Supplementary Fig. 1: Linear regression of relative abundance per taxon (phylum-level) determined via SSU, LSU and combined SSU+LSU. X- and y-axes show relative abundance (%) determined for individual taxa on phylum level using A) SSU and LSU, B) SSU and SSU+LSU, and C) LSU and SSU+LSU as the reference database. Regression analysis showed strong positive relationships (R^2) between each dataset.

Supplementary Table 1: Correlation analysis statistics (SSU, LSU, SSU+LSU). Pearson correlation analysis was performed on relative abundance data (phylum-level) determined via SSU, LSU and SSU+LSU. Correlation coefficients are given in the lower triangle of the matrix, and the two-tailed probabilities that the columns are uncorrelated are given in the upper (PAST v.4.03, Hammer et al., 2001).

Pearson correlation	SSU	LSU	SSU+LSU
SSU	0	2.53E-28	3.49E-27
LSU	0.85105	0	3.28E-49
SSULSU	0.84183	0.94847	0

Supplementary Notes 2: psbO analysis

For an estimate of total abundance of phytoplankton, we ran our data against a recently developed database for the single-copy photosynthetic gene *psbO*, which is present in both prokaryotes and eukaryotes, mainly in one copy per genome[5]. The latter was initially performed with non-subsampled data to be able to determine an adequate subsampling depth[6] (representative of the diversity in our data) for subsequent quantitative analyses (excluding potential artifacts due to differences in library sizes). This non-subsampled data provided a total of 131 *psbO* reads (Supplementary Fig. 2), with no reads identified in libraries that had less than 1.1Mio. raw filtered (post-complexity filtering and deduplication) reads (sample no. 23011, 23019, 23024, 23026, 23075, 23080, 23081, 23143, 23147, 23159, 23162, 23169, Main Text Table 1, Supplementary Fig. 2). Thus, we determined 1.1Mio. reads as an adequate subsampling depth, subsampled all samples to this depth for quantitative *psbO* analyses (main text). The resulting .blastn files of both non-subsampled and subsampled data were converted to .rma6 files using the Blast2RMA tool in MEGAN (version 6_18_9).

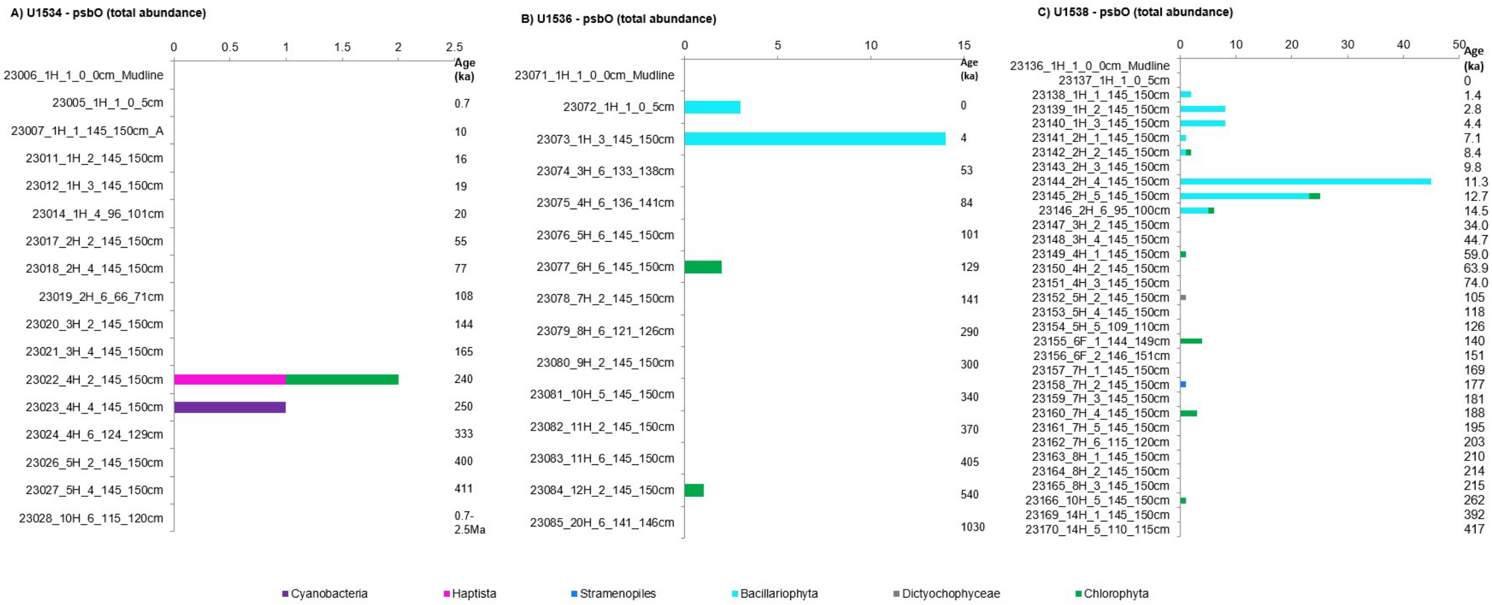

Supplementary Fig. 2: Abundance of photosynthetic organisms at IODP Exp. 382 Sites U1534, U1536, U1538. Abundance of the *psbO* gene (read counts) determined at Exp. 382 Sites U1534 (Hole C) (A), U1536 (Hole B) (B), U1538 (Holes C and D) (C) (phylum-level). Left axis shows the sample identifier, core section and depth, right axis shows the age estimate. Total *psbO* read count: 131 reads (non-standardised raw-data).

We also performed *sed*aDNA damage analysis on both the subsampled and non-subsampled *psbO* data, however, received no output for the subsampled data due to too few input reads (<50[7]). Thus, we only show the *sed*aDNA damage results for the non-subsampled data in Supplementary Data 4.

Supplementary Notes 3: Correlation analyses of sedaDNA damage, taxonomic composition, and geochemical parameters

We used the eukaryote *sed*aDNA damage (%) determined by HOPS post SSU-LSU alignment (Supplementary Data 3), relative abundances of eukaryotes post SSU+LSU alignment (phylum-level), age (ka), the geochemical measurements pH, salinity (‰), alkalinity, phosphate (mM), ammonia (mM), sulfate (SO_4) (mM), and silicon (µM), which were measured as part of the shipboard porewater geochemistry measurements, and downhole formation temperatures that were calculated based on the temperature gradient obtained at each Site with the Advanced Piston Corer Temperature Tool (APCT)[8]. To investigate relationships between taxonomic composition and cold and warm climate phases, we also added benthic $\delta^{18}O$ data from[9] corresponding to the ages assigned to our samples. Mudline samples, as well as the two samples 23162 (U1438C_7H_6_115_120cm) and 23165 (U1538C_8H_3_145_150cm), were removed from the following analyses as no eukaryotes were determined in these samples. Pearson correlation analysis was performed in PAST v.4.03[4].

Correlation analyses revealed positive relationships between *sed*aDNA damage and ammonia (r = 0.56), alkalinity (r = 0.52), phosphate (r = 0.44), $\delta^{18}O$ (r = 0.32), temperature (r = 0.31), and silicone (r = 0.26), very weak positive correlations between *sed*aDNA with pH (r = 0.09), age of sediments (r = 0.02), and negative relationships between *sed*aDNA damage with sulfate (r = -0.42) and salinity (r = -0.15) (Supplementary Fig. 2, Supplementary Data 5). This means that eukaryote *sed*aDNA damage at our sampling locations is primarily associated with indicators for organic matter decomposition, while it is less closely associated with downcore temperature and silicone. Total silicone derived from porewater measurements is expected to be primarily dissolved silica, and hence the weak correlation with eukaryote *sed*aDNA damage might be an indication that diatom DNA in the upper layers might be relatively protected from remineralization until the frustules start to dissolve into silica/silicic acid as they are buried. However, further research is needed into the exact relationships between diatom-specific *sed*aDNA damage, diatom fossil dissolution, dissolved silicon concentrations with depth and associated preservation biases, which are beyond the scope of this study. Correlation analyses between each of the geochemical parameters, as well as benthic $\delta^{18}O$ (from[9]) and the relative abundance of individual eukaryote taxa revealed negative relationships between $\delta^{18}O$ and diatoms (r = -0.41) and positive relationships between $\delta^{18}O$ and Polycystinea (r = 0.42), Dinophycease (r = 0.39) Choanoflagellata (r = 0.38), Mollusca (r = 0.37) and Annelida (r = 0.35), meaning that diatoms were associated with warm phases and dinoflagellates, radiolarian, choanoflagellates and Annelida (includes Crustacea) with cold phases (Supplementary Fig. 2). Relationships between the remaining geochemical parameters and taxonomic composition was random, and data is provided with Supplementary Fig. 3 and Supplementary Data 5.

Supplementary Fig. 3: Correlation analysis plot. Positive correlations are depicted in blue, negative correlations in red, with the size of the circle indicating weak (small) or strong (large) correlation. Significance is depicted by boxes around the circles (i.e., $p<0.05$ is boxed). For details on correlation coefficients between *sed*aDNA damage (SSU+LSU), downhole formation temperature, $\delta^{18}O$ ([9]), geochemical parameters, temperatures, geochemical parameters see Supplementary Data 5.

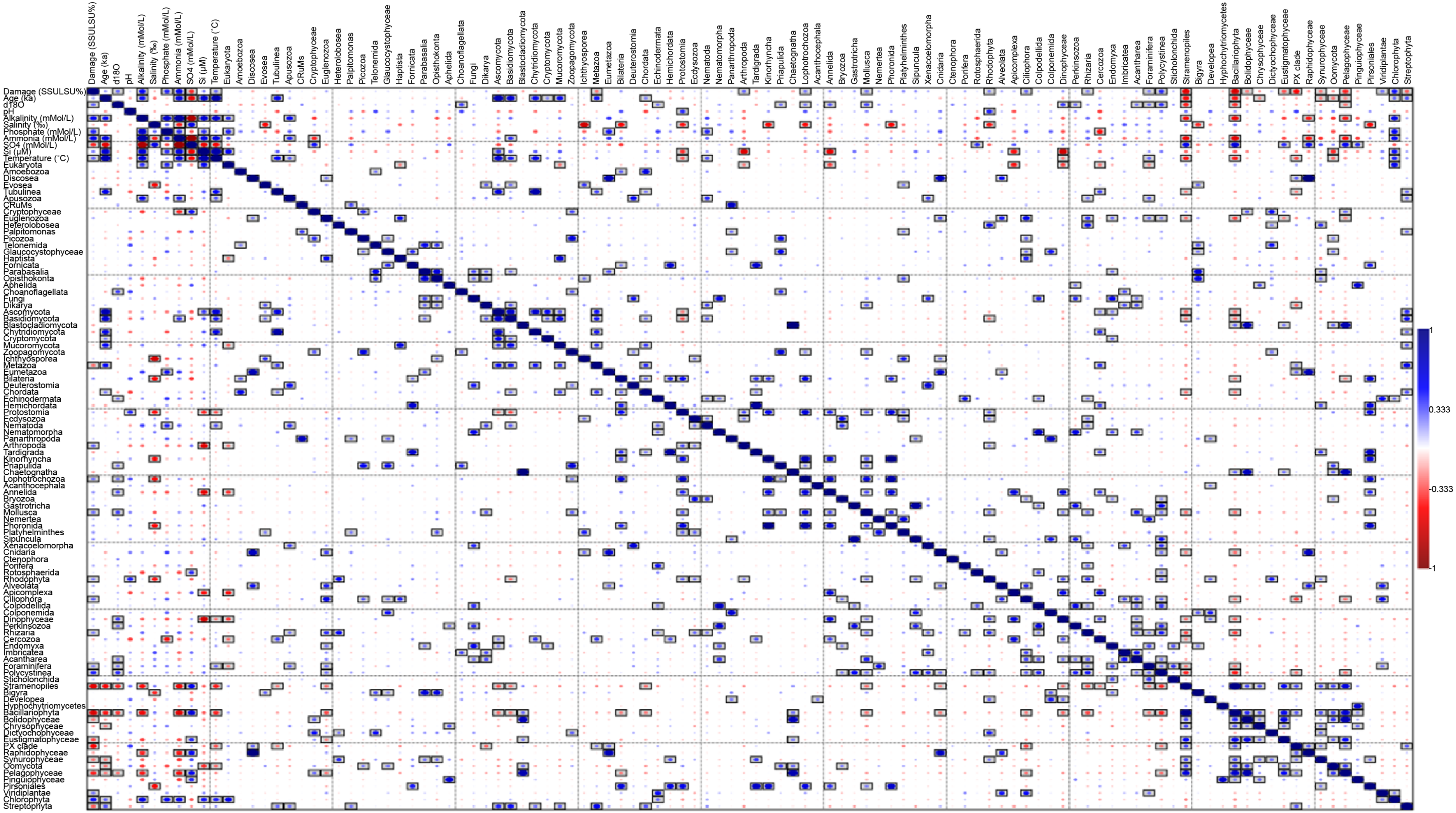

Supplementary References

1. Armbrecht, L. H. The potential of sedimentary ancient DNA to reconstruct past ocean ecosystems. *Oceanography* **33**, 116–123 (2020).

2. Ewels, P., Magnusson, M., Lundin, S. & Käller, M. MultiQC: Summarize analysis results for multiple tools and samples in a single report. *Bioinformatics* **32**, 3047–3048 (2016).

3. Huson, D. H. *et al.* MEGAN Community Edition - Interactive Exploration and Analysis of Large-Scale Microbiome Sequencing Data. *PLoS Comput Biol* **12**, e1004957 (2016).

4. Hammer, Ø., Harper, D. A. T. & Ryan, P. D. PAST: Paleontological Statistics software package for education and data analysis. *Palaeontologia Electronica* **4**, 9 (2001).

5. Pierella Karlusich, J. J. *et al.* A robust approach to estimate relative phytoplankton cell abundance from metagenomic data. *Mol Ecol Resour* PMID35108459 (2022). doi:10.1101/2021.05.28.446125.

6. Cameron, E. S., Schmidt, P. J., Tremblay, B. J. M., Emelko, M. B. & Müller, K. M. Enhancing diversity analysis by repeatedly rarefying next generation sequencing data describing microbial communities. *Sci Rep* **11**, 22302 (2021).

7. Hübler, R. *et al.* HOPS: automated detection and authentication of pathogen DNA in archaeological remains. *Genome Biol* **20**, 1–13 (2019).

8. Weber, M. E. *et al.* Expedition 382 methods. In: Weber, M.E., Raymo, M., Peck, V., Williams, T. & the Expedition 382 Scientists. Iceberg Alley and Subantarctic Ice and Ocean Dynamics. Proceedings of the International Ocean Discovery Program (IODP) 382: College Station, TX (2021). doi:10.14379/iodp.proc.382.102.2021.

9. Lisiecki, L. E. & Raymo, M. E. A Pliocene-Pleistocene stack of 57 globally distributed benthic δ18O records. *Paleoceanography* **20**, PA1003 (2005).
