# Supplementary material for: Ancient marine sediment DNA reveals diatom transition in Antarctica
Source: Nat Commun. 2022 Oct 2;13:5787. doi: 10.1038/s41467-022-33494-4 (PMC9527250; doi:10.1038/s41467-022-33494-4)
Supplement: Supplementary file 2 — Description of Additional Supplementary Files [file 41467_2022_33494_MOESM2_ESM.pdf]

Description of Additional Supplementary Files

File Name: Supplementary Data 1
Description: Perfluoromethyl decaline (PFMD) concentrations. Listed are all PFMD concentrations for inner (core centre) and outer (core periphery, i.e., next to core liner) sediment samples measured by Gas Chromatography. Where no value is given, the corresponding inner and outer samples and measurements were slightly offset in depth (these samples are marked by asterisks). Letters following site names (i.e., A, B, C, D) refer to the site's 'Holes' as per IODP nomenclature (see also3). Values below detection limit are marked as "<DL".

File Name: Supplementary Data 2
Description: Summary of taxa detected via SSU, LSU and SSU+LSU and their representation in the SSU and LSU databases. Where no average abundance value is provided this taxon was not detected via the respective database. Aphelida, Foraminifera, Pirsoniales, Rotosphaerida and Sipuncula were missing in the LSU database and could therefore only be detected via the SSU or SSU+LSU database.

File Name: Supplementary Data 3
Description: Eukaryote sedaDNA damage (%). Details of % eukaryote sedaDNA damage determined by HOPS post SSU-LSU alignment, including average (AV) and standard deviation (SD) per IODP Exp. 382 Site. Samples of Holes C and D at Site U1538 are assigned a "C" and D" in the sample label following the running sample ID number.

File Name: Supplementary Data 4
Description: psbO sedaDNA damage (%). Details of % eukaryote sedaDNA damage determined by HOPS post psbO alignment. Due to very low read numbers even in the non-subsampled data (total of 131 psbO input reads), only samples in which either ancient or default reads were identified are listed, and only for the identified taxa in those samples.

File Name: Supplementary Data 5
Description: orrelation analysis statistics. Pearson correlation values are given in the lower triangle of the matrix, and the two-tailed probabilities that the columns are uncorrelated are given in the upper. Both parametric and non-parametric coefficients and tests are available.

File Name: Supplementary Data 6
Description: Contaminant taxa identified in controls (SSU+LSU). Listed are all taxa identified in Air controls (CtrlAir), PFMD samples (CtrlPFT), and extraction blank controls (EBCs). These taxa were removed from samples and downstream analyses.
