# Supplementary material for: Ancient marine sediment DNA reveals diatom transition in Antarctica
Source: Nat Commun. 2022 Oct 2;13:5787. doi: 10.1038/s41467-022-33494-4 (PMC9527250; doi:10.1038/s41467-022-33494-4)
Supplement: Supplementary file 4 — Reporting Summary [file 41467_2022_33494_MOESM4_ESM.pdf]

nature portfolio

Corresponding author(s): Linda Armbrecht

Last updated by author(s): Sep 3, 2022

Reporting Summary

Nature Portfolio wishes to improve the reproducibility of the work that we publish. This form provides structure for consistency and transparency in reporting. For further information on Nature Portfolio policies, see our Editorial Policies and the Editorial Policy Checklist.

Statistics

For all statistical analyses, confirm that the following items are present in the figure legend, table legend, main text, or Methods section.

n/a	Confirmed	
☐	☒	The exact sample size (*n*) for each experimental group/condition, given as a discrete number and unit of measurement
☒	☐	A statement on whether measurements were taken from distinct samples or whether the same sample was measured repeatedly
☐	☒	The statistical test(s) used AND whether they are one- or two-sided<br>*Only common tests should be described solely by name; describe more complex techniques in the Methods section.*
☒	☐	A description of all covariates tested
☒	☐	A description of any assumptions or corrections, such as tests of normality and adjustment for multiple comparisons
☐	☒	A full description of the statistical parameters including central tendency (e.g. means) or other basic estimates (e.g. regression coefficient) AND variation (e.g. standard deviation) or associated estimates of uncertainty (e.g. confidence intervals)
☐	☒	For null hypothesis testing, the test statistic (e.g. *F*, *t*, *r*) with confidence intervals, effect sizes, degrees of freedom and *P* value noted<br>*Give P values as exact values whenever suitable.*
☒	☐	For Bayesian analysis, information on the choice of priors and Markov chain Monte Carlo settings
☒	☐	For hierarchical and complex designs, identification of the appropriate level for tests and full reporting of outcomes
☐	☒	Estimates of effect sizes (e.g. Cohen's *d*, Pearson's *r*), indicating how they were calculated

Our web collection on statistics for biologists contains articles on many of the points above.

Software and code

Policy information about availability of computer code

Data collection	Physical samples were collected by sediment coring, and sedaDNA extracts and sequencing libraries were prepared physically in a laboratory. The sequencing data analysis is detailed below.
Data analysis	The data was processed and filtered as previously described in detail in:<br>Armbrecht, L. et al. An optimized method for the extraction of ancient eukaryote DNA from marine sediments, Molecular Ecology Resources 20, 906–919 (2020); and Armbrecht, L., et al., Hybridisation capture allows DNA damage analysis of ancient marine eukaryotes, Scientific Reports 11, 3220 (2021).<br>The exact computer code was provided with Armbrecht et al., MER, 2020 (above).<br>Versions of the software used for this manuscript are provided within the Main Manuscript and the Supplementary Information including their references, they include: AdapterRemoval v. 2.1.7-foss-2016a (Schubert et al., 2016); Komplexity version 0.3.6-musl (Clarke et al., 2019); dedupe tool in BBMap v.37.36 (https://jgi.doe.gov/data-and-tools/software-tools/bbtools/.); FastQC v.0.11.4, Babraham Bioinformatics; MultiQC v.1.0.dev0 (Ewels et al., 2016); MALT version 0.4.0 (Herbig et al. 2016); Blast2RMA tool in MEGAN, version 6_18_9 (Huson et al. 2016); MEGAN CE version 6.21.12 (Huson et al., 2016); HOPS v0.33-2 incl. MaltExtract version 1.7 (Hübler, R. et al, 2019); PAST Software v.4.03 (Hammer et al., 2001).

For manuscripts utilizing custom algorithms or software that are central to the research but not yet described in published literature, software must be made available to editors and reviewers. We strongly encourage code deposition in a community repository (e.g. GitHub). See the Nature Portfolio guidelines for submitting code & software for further information.

Data

Policy information about availability of data

All manuscripts must include a data availability statement. This statement should provide the following information, where applicable:

- Accession codes, unique identifiers, or web links for publicly available datasets
- A description of any restrictions on data availability
- For clinical datasets or third party data, please ensure that the statement adheres to our policy

The following databases were used in this study: SILVA small (version 132Ref-nr) and large (version 132Ref) subunit ribosomal RNA database (https://www.arb-silva.de/), and psbO53 (https://www.ebi.ac.uk/biostudies/studies/S-BSST659?query=S-BSST659). Source data are provided with this paper. Detailed Supplementary Information on methods and analysis is provided with this submission. The demultiplexed raw sequencing data generated and analysed during this study have been deposited in the NCBI Sequence Read Archive database (https://www.ncbi.nlm.nih.gov/sra) under Accession code/BioProject PRJNA861836 (https://www.ncbi.nlm.nih.gov/bioproject/PRJNA861836/; BioSamples SAMN29928044 - SAMN29928123), and includes metadata for each sediment and control sample. For further requests please contact the corresponding author.

Field-specific reporting

Please select the one below that is the best fit for your research. If you are not sure, read the appropriate sections before making your selection.

☐ Life sciences ☐ Behavioural & social sciences ☒ Ecological, evolutionary & environmental sciences

For a reference copy of the document with all sections, see nature.com/documents/nr-reporting-summary-flat.pdf

Ecological, evolutionary & environmental sciences study design

All studies must disclose on these points even when the disclosure is negative.

Study description

We present authenticated (through extensive contamination control and sedaDNA damage analysis) metagenomic marine eukaryote sedaDNA from the Scotia Sea region acquired during IODP Expedition 382. We also provide a marine eukaryote sedaDNA record of ~1 Mio. years and diatom and chlorophyte sedaDNA dating back to ~540 ka (using taxonomic marker genes SSU, LSU, psbO). We find evidence of warm phases being associated with high relative diatom abundance, and a marked transition from diatoms comprising <10% of all eukaryotes prior to ~14.5 ka, to ~50% after this time, i.e., following Meltwater Pulse 1A, alongside a composition change from sea-ice to open-ocean species. Our study demonstrates that sedaDNA tools can be expanded to hundreds of thousands of years, opening the pathway to the study of ecosystem-wide marine shifts and paleo-productivity phases throughout multiple glacial-interglacial cycles. The study is based on a total of 80 sedaDNA extracts/metagenomic shotgun libraries from 65 sediment samples, 6 air controls, 3 PFMD controls, and 6 extraction blank controls (see Data collection below).

Research sample

Marine sediments collected via deep ocean sediment coring (see Sampling Strategy below). These samples include the ancient DNA of bacteria, archaea and eukaryota; the eukaryote portion is the focus of this research, they were not manipulated other than applying a sedaDNA extraction protocol that is optimised for the extraction of marine eukaryote sedaDNA (see Data collection below). The sediment samples are dated from present to ~1 million years (see Dating methods below).

Sampling strategy

Sediment samples were collected during IODP Exp. 382 'Iceberg Alley and Subantarctic Ice and Ocean Dynamics' on-board RV Joides Resolution between 20 March and 20 May 2019. Specifically, we collected samples at Site U1534 (Falkland Plateau, 606 m water depth), U1536 (Dove Basin, Scotia Sea, 3220 m water depth), and Site U1538 (Pirie Basin, Scotia Sea, 3130 m water depth). We used advanced piston coring (APC) to acquire sediment cores, which recovers the least disturbed sediments.

All sediment samples were taken on the ship's 'catwalk', where, once the core was on deck, the core liners were wiped clean twice (3% sodium hypochlorite, 'bleach') at each cutting point. Core cutting tools were sterilised before each cut (3% bleach and 80% ethanol) of the core in 1m sections. The outer ~3 mm of surface material were removed from the bottom of each core section to be sampled, using sterilised scrapers (~4 cm wide; bleach and ethanol treated). A cylindrical sample was taken from the core centre using a sterile (autoclaved) 10 mL cut-tip syringe, providing ~5 cm3 of sediment material. The syringe was placed in a sterile plastic bag (Whirl-Pak) and immediately frozen at −80°C. The mudline (sediment/seawater interface) was transferred from the core liner into a sterile bucket (3% bleach treated), and 10 mL sample was retained in a sterile 15 mL centrifuge tube (Falcon) and frozen at −80°C. Samples were collected at various depth intervals depending on the site to span the Holocene up to ~1 million years (Table 1). This lower depth/age limit was determined by switching coring system from APC to the extended core barrel (XCB) system.

To test for potential airborne contamination, two air control was taken during the sedaDNA sampling process per site. For this, an empty syringe was held for a few seconds in the sampling area and then transferred into a sterile plastic bag and frozen at -80 °C. In total, we collected 65 sediment samples, and 6 air controls.

To assess the potential for contamination due to drill fluid making contact with the core liner, we added the non-toxic chemical tracer perfluoromethyldecalin (PFMD) to the drill fluid at a rate of ~0.55 mL min-1 for cores collected at Sites U1534 and U1536. As we found that PFMD concentrations were very low at these sites, the infusion rate was doubled prior to sedaDNA sampling at Site U1538 to ensure low PFMD concentrations represent low contamination and not delivery failure of PFMD to the core. At each sedaDNA sampling depth, one PFMD sample was taken from the periphery of the core (prior to scraping, to test whether drill fluid reached the core pipe), and one next to the sedaDNA sample in the centre of the core (after scraping, to minimise differences to the sedaDNA sample, and testing if drill fluid had reached the core centre). We transferred ~3 cm3 of sediment using a disposable, autoclaved 5 mL cut-tip syringe into a 20 mL headspace vial with metal caps and Teflon seals. We also collected a sample of the tracer-infused drill fluid at each site, by transferring ~10 mL of the fluid collected at the injection pipe on the rig floor via a sterile plastic bottle into a 15 mL centrifuge tube (inside a sterile plastic bag) and freezing it at -80°C. Samples were analysed using gas chromatography (GC-µECD;

Hewlett-Packard 6890).
A detailed description of the PFMD GC measurements is provided in Weber et al., Proceedings of the IODP Exp 382, 2021. Briefly, PFMD measurements were undertaken in batches per site for U1534, U1536, and U1538. This included the analyses of PFMD samples collected at two additional holes at these sites, U1534D and U1536C, from which we also collected sedaDNA samples but that are not part of this study. PFMD is categorized as the stereoisomers of PFMD (C11F20), which add up to 87-88% (and with the remaining 12% being additional perfluoro compounds unable to be separated by the manufacturer). We exclusively refer to the first and measurable PFMD category, calibrating for the 88% in bottle concentrations during concentration calculations. Each GC analysis run included the measurement of duplicate blanks and duplicate PFMD standards. Due to a large sample number, PFMD at Site U1538 was measured in three separate runs, with the first and last run including triplicate blank and triplicate PFMD standards (duplicates in the second run), and the last run also containing a drill-fluid sample. To blank-correct PFMD concentrations, we subtracted the average PFMD concentration of all blanks per run from PFMD measurements in that run. To determine the detection limit of PFMD, we used three times the standard deviation of the average blank PFMD values per run; due to all blank values for the U1538 runs being 0, we used three times the standard deviation of the lowest PFMD standard for this site in this calculation. This provided us with a PFMD detection limit of 0.2338 ng mL-1. Any PFMD measurements of samples below this limit were rejected.

Data collection

A total of 80 sedaDNA extracts and metagenomic shotgun libraries were prepared following Armbrecht et al., MER, 2020, and Armbrecht et al. Sci.Rep., 2021. This included 65 sediment samples, 6 air controls, 3 PFMD controls, and 6 extraction blank controls (see below). We randomised our samples and controls and extracted sedaDNA in batches of 16 extracts/libraries at a time, with each batch including at least one air control and one extraction blank control (EBC), and the last batch including mudline and PFMD samples to avoid contamination of the sedaDNA samples. A complete list of sedaDNA samples and controls is provided in the manuscript (Table 1). The laboratory procedure was documented by the corresponding author in digital and paper-based protocols and notes. The libraries sequenced at the Garvan Institute for Medical Research, Sydney, Australia (Illumina NovaSeq 2 x 100 bp).

Timing and spatial scale

The timing of the sample collection for this study was determined by the dates scheduled for IODP Exp. 382, which was from 20 March to 20 May 2019. Samples at Site U1534 were collected on 01 April 2019, at Site U1536 on 12-13 April, 2019, and at Site U1538 on 11 (U1538C) and 13 (U1538D) May 2019.
Site U1534 (53°11.3865'S, 58°45.6296'W) is located at the Subantarctic Front contourite drift at the northern limit of the Scotia Sea, and Sites U1536 (59°26.4608'S, 41°3.6399'W) and U1538 (57°26.5387'S, 43°21.4521'W) are located in the southern and central Scotia Sea, respectively.

Data exclusions

PFMD data: Any PFMD measurements of samples below the detection limit (0.2338 ng mL-1) were rejected/excluded.
sedaDNA data: Reads for species identified in EBCs, air, and PFMD controls were subtracted from sediment sample setaDNA data, which was conducted with MEGAN CE (version 6.21.12). All taxa determined in the controls are provided with the Supplementary Data.

Reproducibility

Our dataset is unique, and was generated over a total of 3 years including sediment coring of the deep seafloor in remote Antarctica, laboratory work, and bioinformatic processing, and analysis. It was generated using the most stringent, recommended anti-contamination procedures. During the laboratory phase samples were randomised, and 20% of the dataset included controls. Therefore, we have not repeated this study.

Randomization

We randomised our samples and controls and extracted sedaDNA in batches of 16 extracts/libraries at a time, with each batch including at least one air control and one extraction blank control (EBC), and the last batch including mudline and PFMD samples to avoid contamination of the sedaDNA samples.

Blinding

During the sedaDNA extraction and library preparation process, we labelled the samples with running numbers from 1-16 (16 samples per batch). This means there were no biases as to what sample was a sediment sample, control, specific site, or specific age. Bioinformatic processing was performed the same way for all samples and controls using the same commands, thus biases in the computational analyses were impossible.

Did the study involve field work? ☒ Yes ☐ No

Field work, collection and transport

Field conditions

Field work included sediment coring of the deep ocean in remote Antarctica. Generally, sediment coring is only possible in relatively calm seas. Upon retrieval, sediment cores were immediately transported to an indoors area (the 'catwalk') nearby, where core cutting and sampling under clean conditions (described above) took place. The catwalk is open to the outdoors at one end, i.e., sampling occurred under normal, cool air conditions characteristic for the study region, with no exposure to precipitation.

Location

Site U1534 (53°11.3865'S, 58°45.6296'W, water depth 606.27m) is located at the Subantarctic Front contourite drift at the northern limit of the Scotia Sea, and Sites U1536 (59°26.4608'S, 41°3.6399'W, water depth 3220.06m) and U1538 (57°26.5387'S, 43°21.4521'W, water depth 3130.21m) are located in the southern and central Scotia Sea, respectively.
Samples at Site U1534 were collected on 01 April 2019, at Site U1536 on 12-13 April, 2019, and at Site U1538 on 11 (U1538C) and 13 (U1538D) May 2019.

Access & import/export

IODP Expedition proposals undergo a rigorous environmental protection and safety review, which is approved by the IODP's Environmental Protection and Safety Panel (EPSP) and/or the Safety Panel. The same procedure was applied to IODP Exp. 382 and approval was provided by the EPSP. Sediment samples for sedaDNA analyses were imported to Australia under Import Permit number 0002658554 provided by the Australian Government Department for Agriculture and Water Resources (date of issue: 19 September 2018), and were stored and extracted at a quarantine approved facility (AA Site No. S1253, Australian Centre for Ancient DNA).

Disturbance

Neither fieldwork, lab work or bioinformatic data processing caused any disturbances.

Reporting for specific materials, systems and methods

We require information from authors about some types of materials, experimental systems and methods used in many studies. Here, indicate whether each material, system or method listed is relevant to your study. If you are not sure if a list item applies to your research, read the appropriate section before selecting a response.

Materials & experimental systems

n/a	Involved in the study
☒	☐ Antibodies
☒	☐ Eukaryotic cell lines
☐	☒ Palaeontology and archaeology
☒	☐ Animals and other organisms
☒	☐ Human research participants
☒	☐ Clinical data
☒	☐ Dual use research of concern

Methods

n/a	Involved in the study
☒	☐ ChIP-seq
☒	☐ Flow cytometry
☒	☐ MRI-based neuroimaging

Palaeontology and Archaeology

Specimen provenance

Metagenomics ancient DNA analyses were performed on sediment samples. Sediment samples were imported to Australia under Import Permit number 0002658554 provided by the Australian Government Department for Agriculture and Water Resources (date of issue: 19 September 2018), and stored and extracted at a quarantine approved facility (AA Site No. S1253, Australian Centre for Ancient DNA).

Specimen deposition

The sediment samples are now stored at the quarantine approved facilities of the Institute for Marine and Antarctic Studies (IMAS), University of Tasmania (AA Site No. T1325), following Sample Transfer Approval T2021/008 provided by from the Australian Government Department of Agriculture Water and Environment. Subsamples can be requested from the corresponding author Dr L. Armbrecht upon reasonable request and in accordance with IODP guidelines.

Dating methods

Age control for Site U1534 is based on tuning of benthic foraminifera d18O to the LR04 stack (Peck & Alcock, NERC EDS UK Polar Data Centre, 2022). Wherever present specimens of Uvigerina bifurcata were picked from samples at 10 cm intervals. During warmer periods when U. bifurcata was not present, Melonis affinis and/or Hoeglundina elegans were analysed. Sedimentation rates over the intervals sampled for sedaDNA typically range between 6 and 30 cm/kyr, with rates exceeding 100 cm/kyr during the Last Glacial Maximum approximately 20,000 years ago (20 ka). For our deepest sample, U1534C-10H-6_115cm (90.95 mbsf), we only have biostratigraphically assigned ages available (shipboard data), which date this sample as early Pleistocene (~2.5 – 0.7 million years ago, Ma, Peck et al., IODP Proceedings Site U1534, 2021).

Low-resolution age control for both Sites U1536 and U1538 was established using shipboard magneto- and biostratigraphy (Reilly et al., Paleoceanography and Paleoclimatology, 2021; Weber et al., Proceedings IODP Exp. 382, 2021). Average sedimentation rates are ~10 cm/kyr for Site U1536, with elevated values (up to 20 cm/kyr) in the upper ~80 mbsf (the last ~400 ka). Site U1538 average sedimentation rates are twice as high, averaging ~20 cm/kyr. Especially in the upper ~430 mbsf (the last 1.8 Ma), rates are up to 40 cm/kyr. Higher resolution age models are based on dust climate couplings, correlating sedimentary dust proxy records such as magnetic susceptibility and sedimentary Ca and Fe records to ice-core dust proxy records over the last 800 ka (Weber et al., Nat Commun 13:2044, 2022) and to a benthic isotopic stack (Lisiecki & Raymo, Paleoceanography, 2005) before that. These age models were established for Site U1537 (adjacent to Site U1536) and provide orbital to millennial scale resolution. For this study we correlated sedimentary cycles of Sites U1536 and U1538 to U1537 to achieve similar resolution and to be able to determine if a sample originates from a glacial or interglacial period (Main Text Table 1). The ages are provided within the main manuscript.

☒ Tick this box to confirm that the raw and calibrated dates are available in the paper or in Supplementary Information.

Ethics oversight

No ethical approval was required for this study. Metagenomic analyses of deep seafloor samples are not expected to contain sensitive information. Additionally, our studies' focus is on photosynthetic microalgae, key indicators of climate change, but of no ethical concern.

Note that full information on the approval of the study protocol must also be provided in the manuscript.
